# Assembly of nitrogenase biosynthetic pathway in *Saccharomyces cerevisiae* by using polyprotein strategy

**DOI:** 10.3389/fmicb.2023.1137355

**Published:** 2023-03-02

**Authors:** Minyang Wang, Yimin Shang, Xiaomeng Liu, Sanfeng Chen

**Affiliations:** State Key Laboratory for Agrobiotechnology and College of Biological Sciences, China Agricultural University, Beijing, China

**Keywords:** nitrogenase, 2A peptide, polyprotein, co-expression, *Saccharomyces cerevisiae*

## Abstract

Nitrogenase in some bacteria and archaea catalyzes conversion of N_2_ to ammonia. To reconstitute a nitrogenase biosynthetic pathway in a eukaryotic host is still a challenge, since synthesis of nitrogenase requires a large number of *nif* (*ni*trogen *f*ixation) genes. Viral 2A peptide mediated “cleavage” of polyprotein is one of strategies for multigene co-expression. Here, we show that cleavage efficiency of NifB-2A-NifH polyprotein linked by four different 2A peptides (P2A, T2A, E2A, and F2A) in *Saccharomyces cerevisiae* ranges from ~50% to ~90%. The presence of a 2A tail in NifB, NifH, and NifD does not affect their activity. Western blotting shows that 9 Nif proteins (NifB, NifH, NifD, NifK, NifE, NifN, NifX, HesA, and NifV) from *Paenibacillus polymyxa* that are fused into two polyproteins *via* 2A peptides are co-expressed in *S*. *cerevisiae*. Expressed NifH from *Klebsiella oxytoca* NifU and NifS and *P*. *polymyxa* NifH fusion linked *via* 2A in *S*. *cerevisiae* exhibits Fe protein activity.

## Introduction

Although nitrogen is abundant in the atmosphere as N_2_ on earth, the N_2_ is not easily available to living organisms, and the bio-available nitrogen (such as ammonia and nitrogen oxides) limits the productivity of major crops (Hoffman et al., 2014). The addition of industrially produced nitrogenous fertilizer can maintain crop productivity, but overuse of chemical nitrogen fertilizers leads to economic costs and environmental pollution (Zhang et al., 2015). One way to reduce use of fertilizers is to transfer biological nitrogen fixation to cereal crops that can fix nitrogen (Beatty and Good, 2011; Curatti and Rubio, 2014; Oldroyd and Dixon, 2014; Vicente and Dean, 2017; Good, 2018; Li and Chen, 2020).

Biological nitrogen fixation, mainly carried out by Mo nitrogenase enzyme in some bacteria and archaea, is a process in which atmospheric N_2_ is converted to ammonia (Dos et al., 2012; He et al., 2022). Nitrogenase consists of two metalloprotein components: Fe protein and MoFe protein (Georgiadis et al., 1992). Fe protein, also termed as NifH protein, is a homodimer (encoded by *nifH*) bridged by an intersubunit [4Fe-4S] cluster that serves as the obligate electron donor to the MoFe protein. MoFe protein, also termed as NifDK protein, is a heterotetramer (encoded by *nifD* and *nifK*) that contains two metalloclusters: FeMo-cofactor (Mo − 7Fe−9S − C − homocitrate) that serves as the active site of substrate binding and reduction, and the P cluster (8Fe−7S) that shuttles electrons to FeMo-co (Burén et al., 2020). Apart from *nifH*, *nifD* and *nifK* encode the structural subunits of nitrogenase, several genes are required for the biosynthesis of the metalloclusters. *nifU* and *nifS* are involved in synthesis of Fe-S cluster (Dos Santos et al., 2007). *nifF* and *nifJ* are involved in transport of electron. *nifE*, *nifN*, *nifX*, *nifB*, *nifQ*, *nifV*, *nifY*, and *nifH* contribute to the synthesis and insertion of FeMo-co into nitrogenase, and *nifM* is required for proper folder of nitrogenase Fe protein (Roberts et al., 1978; Gavini et al., 2006; Rubio and Ludden, 2008; Hu and Ribbe, 2011). The *nif* genes required for synthesis of nitrogenase vary greatly amongst species. For example, in *Klebsiella oxytoca*, 20 *nif* genes (*JHDKTYENXUSVWZMFLABQ*) are co-located within a ∼ 24 kb cluster (Arnold et al., 1988). Whereas, *Paenibacillus polymyxa* WLY78 has a minimal *nif* gene cluster composed of nine genes (*nifBHDKENXhesAnifV*). The *nif* gene cluster from *P*. *polymyxa* WLY78 enabled *Escherichia coli* to synthesize active nitrogenase (Wang et al., 2013). The four genes (*nifS*, *nifU*, *nifF* and *nifJ*) from *K*. *oxytoca* could enhance the nitrogenase activity of the recombinant *Escherichia coli* 78–7 carrying the *P*. *polymyxa nif* cluster (Li et al., 2016).

*Saccharomyces cerevisiae*, a eukaryotic organism, is widely used as a host for heterologous expression of metabolite biosynthetic pathway. Recently, several *nif* genes from different diazotrophic bacteria and archaea have been successfully expressed in *S*. *cerevisiae*. The active NifH was generated in *S*. *cerevisiae* by expressing the 4 genes *nifH*, *nifU*, *nifS*, and *nifM* from *Azotobacter vinelandii*. Of the four genes, *nifH* and *nifM* are cloned in one plasmid vector, and *nifS* and *nifU* are cloned in another plasmid vector with each gene being under control of a promoter and then the two recombinant plasmids were co-transformed into yeast to express the four Nif proteins (López-Torrejón et al., 2016). The NifDK tetramer with a smaller molecular weight than that of *A*. *vinelandii* was formed in *S*. *cerevisiae* by expressing the 9 genes (*nifH*, *nifD*, *nifK*, *nifU*, *nifS*, *nifM*, *nifE*, *nifN*, and *nifB*) from *A*. *vinelandii* (Buren et al., 2017). To express the nine *nif* genes in yeast, constraint-based combinatorial design and DNA assembly are used to build libraries of *nif* gene clusters, and then promoters and terminators were combined *to the nine nif genes to generate nine transcript units via Type IIS assembly (*Weber et al., 2011*)*. *The nine transcription units were assembled into three subclusters that contain homologous sequences to one another and to the S*. *cerevisiae genome*. *Finally*, *nine nif genes (HDKUSMBEN) were integrated into the yeast genome by homologous recombination when three subclusters were transformed into yeast* (Buren et al., 2017). An [8Fe-9S-C] cluster, called NifB-co, which constitutes the core of the FeMo-co (Mo − 7Fe−9S − C − homocitrate), was formed in yeast by co-expressing *Methanocaldococcus infernus nifB* together with *nifU*, *nifS*, and *fdxN* from *A*. *vinelandii* (Burén et al., 2019). Our recent study also has shown that the active NifH and the NifDK tetramer were produced in *S*. *cerevisiae* by engineering 15 gene (*nifB*, *nifH*, *nifD*, *nifK*, *nifE*, *nifN*, *nifX*, *hesA*, *nifV*, *groES* and *groEL* from *P*. *polymyxa* WLY78 and 4 genes *nifS*, *nifU*, *nifF*, and *nifJ* from *K*. *oxytoca* (Liu et al., 2019). Each of the 15 genes was fused with a promoter and a terminator of *S*. *cerevisiae* to generate 15 transcription units by using overlap extension PCR. The 11 transcription units (*nifB*, *nifH*, *nifD*, *nifK*, *nifE*, *nifN*, *nifX*, *hesA*, *nifV*, *groES*, and *groEL*) containing *homologous sequences to one another and to the S*. *cerevisiae genome were integrated into the δ site of S*. *cerevisiae chromosome in a one-step fashion and the* 4 transcription units (*nifS*, *nifU*, *nifF*, and *nifJ*) were integrated onto the *HO* site of the *S*. *cerevisiae* chromosome (Liu et al., 2019).

Biological nitrogen fixation requires a large number of *nif* genes. How to engineer large numbers of *nif* genes into eukaryotic organism is still a challenge. Co-expression of multiple genes at a desired ratio is one strategy to solve the challenge. 2A peptides, the “self-cleaving” small (18–22 amino acids) peptides, are used to express multiple proteins from a single open reading frame (ORF) in eukaryotic cells (Ryan et al., 1999). When two genes are linked *via* a 2A peptide sequence, two separate proteins are generated from an ORF through a novel “cleavage” event within the 2A peptide sequence (Atkins et al., 2007; Doronina et al., 2008). Cleavage occurs at the end of the 2A peptide sequence through translation by 80S ribosomes (Szymczak-Workman et al., 2012). The cleavage of 2A peptides is not associated with the host cell proteases or viral proteinases, instead, associated with the highly conserved C-terminal of the 2A peptides (-DxExNPGP-) (Palmenberg et al., 1992; Donnelly et al., 2001; Sharma et al., 2012). 2A peptides were first discovered in the foot-and-mouth disease virus (FMDV) (Ryan and Drew, 1994). Since then, many 2A-like sequences have been identified in other viruses (Szymczak and Vignali, 2005; Wang and Marchisio, 2021). Four 2A peptides have been widely used in biomedical research: FMDV 2A (abbreviated herein as F2A); equine rhinitis A virus (ERAV) 2A (E2A); porcine teschovirus-1 2A (P2A) and thosea asigna virus 2A (T2A).

In this study, cleavage efficiency of NifB and NifH proteins linked *via* four different 2A peptides in *S*. *cerevisiae* are investigated by using Western blotting and yeast two-hybrid system analysis. Then, nine Nif proteins (NifB, NifH, NifD, NifK, NifE, NifN, NifX, HesA, and NifV) from *P*. *polymyxa* WLY78 are fused into two huge proteins and then they are co-expressed in *S*. *cerevisiae*. *P*. *polymyxa* NifH and *K*. *oxytoca* NifS and NifU are fused into a polyprotein and co-expressed in *S*. *cerevisiae*, and the expressed NifH from the huge protein (NifS-2A-NifU-2A-NifH) exhibits Fe activity. This study shows the potential of utilization of 2A peptides to express multicomponent nitrogenase system in *S*. *cerevisiae*.

## Results and discussion

### Cleavage efficiency of NifB and NifH proteins linked *via* 2A peptide in yeast

**Figure 1:**
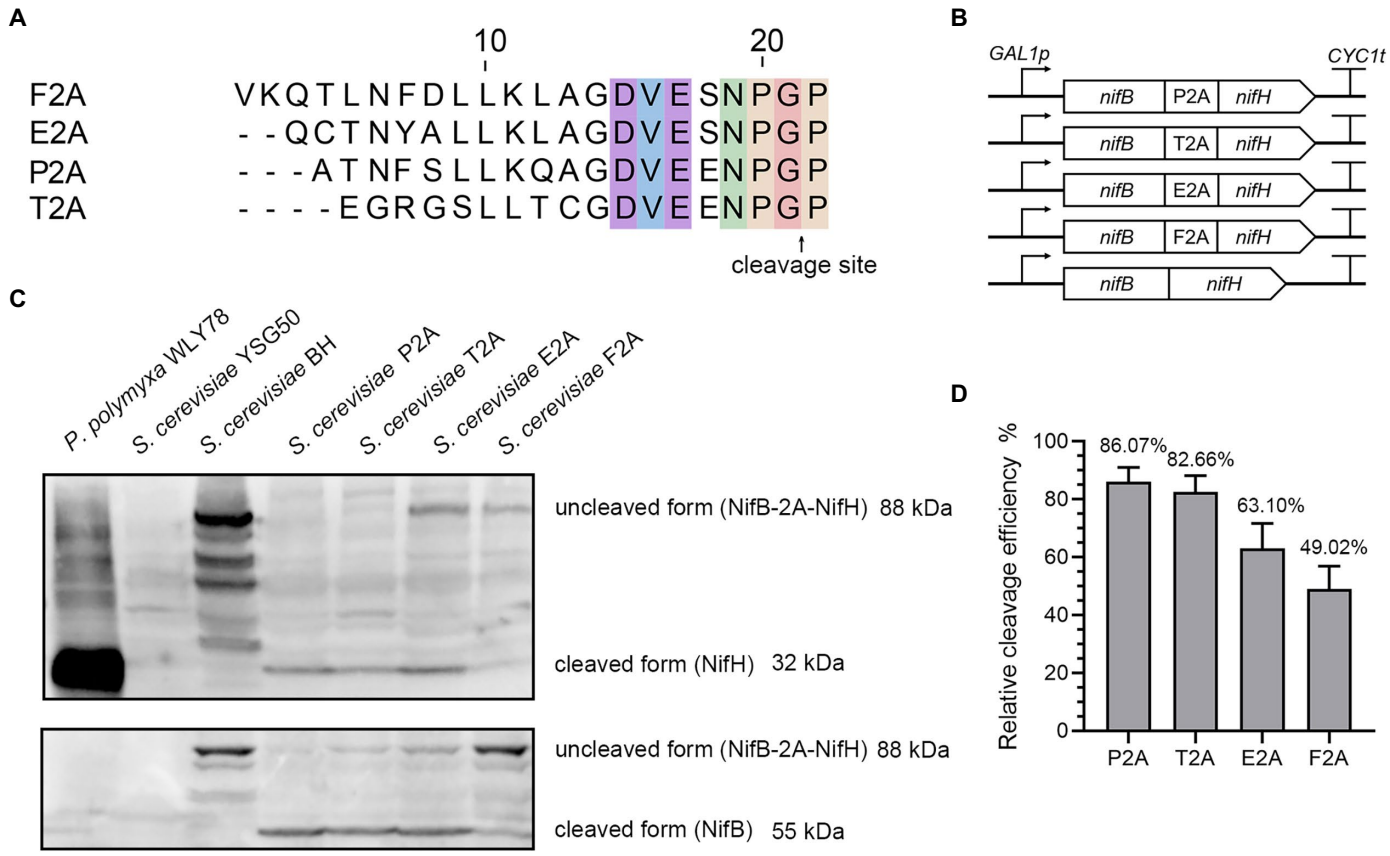
The cleavage efficiency of NifB and NifH linked *via* four different 2A sequences. **(A)** Amino acid sequences of the 2A peptides used in this study. **(B)** Schematic diagrams of the constructs of *nifB* and *nifH* linked *via* 4 different 2A peptides and without 2A peptide in the recombinant vectors. *GAL1p* indicates promoter and *CYC1t* indicates terminator. **(C)** Western blotting analysis of cell homogenates using antibody against NifH and NifB, respectively. *P*. *polymyxa* WLY78 was used as a positive control. The recipient *S*. *cerevisiae* YSG50 and *S*. *cerevisiae* BH carrying NifB-NifH fusion protein without 2A peptide linker are used as negative controls. P2A, T2A, E2A, and F2A indicate that *S*. *cerevisiae* strains carrying NifB-NifH fusion protein linked *via* P2A, T2A, E2A, and F2A, respectively. **(D)** Quantitation of the cleavage efficiency of NifB and NifH mediated by different 2A sequences. Cleavage efficiency = NifH/ (NifH+NifB-2A-NifH) *100%. The band intensity on the Western blot was measured by ImageJ software. Error bars indicated SEM, *n* = 3.

To test whether 2A peptide was able of separating bacterial nitrogenase proteins, two Nif proteins (NifB and NifH), which are the first and second proteins in Nif-cluster (NifBHDKENXHesANifV) of *P*. *polymyxa* WLY78, were chosen to be tested (Wang et al., 2013). The *nifB* and *nifH* genes, codon optimized for *S*. *cerevisiae*, were linked into a single open reading frame (ORF) encoding a polyprotein (designated NifB-2A-NifH) by four different 2A peptides (P2A, T2A, E2A and F2A), respectively (Figure 1A). Then, each of a single ORF was cloned into pRS423-*GAL1p* plasmid under the control of *GAL1* promoter (Figure 1B), and each plasmid was transformed into *S*. *cerevisiae* YSG50. To determine the cleavage efficiency of the 2A peptide linked NifB and NifH, total protein extracts were prepared from aerobically grown yeast cultures and analyzed by Western blotting with the antibody against the NifH and NifB proteins of *P*. *polymyxa* WLY78 which was expressed and purified from *E*. *coli* BL21. The calculated molecular weights of NifH and NifB were 31.5 KDa and 55.0 KDa, respectively. The NifH protein was detectable in the recombinant *S*. *cerevisiae* strains but not in the wild-type *S*. *cerevisiae* YSG50 (Figure 1C). All the four 2A peptides evaluated showed cleavage with different efficiency, ranging from ~50% to ~90%. The best performance was achieved using P2A and T2A, in which the cleaved form represented more than 80% (Figure 1D). Our data are consistent with the reports that order of the work efficiency is P2A, T2A, F2A, and E2A (Szymczak-Workman et al., 2012). The results indicate that P2A and T2A should be preferred for expression of Nif proteins in yeast.

In addition, the different cleavage efficiency among the four 2A peptides was verified by yeast two hybrid assay *in vivo*. Two vectors pGBKT7 carrying *GAL4* DNA-binding domain (BD) and pGADT7 carrying a *GAL4* activation domain (AD) are usually used in yeast two hybrid (Supplementary Figure S1A). Here, a middle vector pGBKT7-AD that simultaneously carries AD and BD of *GAL4* was constructed by cloning the *GAL4* activation domain (AD) to vector pGBKT7 as described in Materials and Methods. Then, five *nifB* and *nifH* fusions linked with four different 2A peptides or without 2A peptide were individually in frame inserted between BD and AD domains of *GAL4* in vector pGBKT7-AD, and then *GAL4 BD*, *nifB*, *nifH and GLA4 AD* were fused into a single ORF under control of *ADH1* promoter (Supplementary Figure S1B). These plasmids were individually transformed into *S*. *cerevisiae* YSG50, yielding five recombinant strains: *S*. *cerevisiae* Y2H-BH carrying NifB-NifH, *S*. *cerevisiae* Y2H-BP2AH carrying NifB-P2A-NifH, *S*. *cerevisiae* Y2H-BT2AH carrying NifBT2ANifH, *S*. *cerevisiae* Y2H-BE2AH carrying NifH-E2A-NifH and *S*. *cerevisiae* Y2H-BF2AH carrying NifB-F2A-NifH (Supplementary Table S1). Yeast strain Y2H Gold carrying vector pGBKT7 was used as a negative control. All yeast strains grew well on the SC-Trp plates (Supplementary Figure S1C), indicating that TRP1 carried in vector pGBKT7 was expressed. When grown on SC-Trp-His-Ade plates, *S*. *cerevisiae* BH carrying NifB-NifH grew well, suggesting that *GAL4* AD-NifB-NifH-*GAL4* BD was a polyprotein. Whereas, *S*. *cerevisiae* BP2AH carrying NifB-P2A-NifH, *S*. *cerevisiae* BT2AH carrying NifBT2ANifH, *S*. *cerevisiae* BE2AH carrying NifH-E2A-NifH could not grow on SC-Trp-His-Ade plates, suggesting that NifB and NifH was effectively separated by P2A, T2A and E2A. However, *S*. *cerevisiae* BE2AH carrying NifH-F2A-NifH could grow well as *S*. *cerevisiae* BH carrying NifB-NifH did on SC-Trp-His-Ade plates, suggesting that E2A could not effectively separate NifB and NifH. The results are consistent with the western blotting data described above.

### Tailing-tolerance assay

Since the 2A tail remains attached to the carboxyl terminus of the upstream protein after cleavage, it necessary to investigate influence of the presence of a 2A tail on Nif proteins. Here, the tolerance to the presence of a 2A tail is determined by using Nif protein carrying a 2A tail to complements nitrogenase activity of Δ*nif* mutant of *P*. *polymyxa*. As described in Materials and Methods, *nifH* gene carrying four different 2A peptides (P2A, T2A, E2A and F2A) was used to complement Δ*nifH* mutant of *P*. *polymyxa*, generating four complementation strains *P*. *polymyxa* HPtail, *P*. *polymyxa* HTtail，*P*. *polymyxa* HEtail and *P*. *polymyxa* HFtail. *P*. *polymyxa* proH carrying *nifH* without 2A tail was constructed by using *nifH* gene without 2A tail to complement Δ*nifH* mutant of *P*. *polymyxa* was used as a positive control. Nitrogenase activity analyses showed that strains carrying NifH with different 2A tail exhibited the similar nitrogenase activities as *P*. *polymyxa* proH carrying *P*. *polymyxa* NifH without 2A tail did (Supplementary Figure S2). The data suggest that the 2A tails did not affect activity of NifH protein.

Further, we selected P2A, an efficient cutter, to verify its influence on the three major nitrogenase proteins NifB, NifD, and NifK. As described in Materials and Methods, complementation strains *P*. *polymyxa* Btail, *P*. *polymyxa* Dtail and *P*. *polymyxa* Ktail were constructed by using *nifB*, *nifD* and *nifK* carrying P2A tail to complement Δ*nifB*, Δ*nifD*, Δ*nifK* mutants of *P*. *polymyxa*, respectively. *P*. *polymyxa* proB, *P*. *polymyxa* proD and *P*. *polymyxa* proK that contained, respectively, *nifB*, *nifD*, and *nifK* without P2A were used as positive controls. Nitrogenase activity analyses showed that the residual P2A tail had no effect on the activity of NifB and NifD, while P2A tail made NifK to lose activity (Supplementary Figure S2B). The result is consistent with the reports that ENLYFQ tail on *K*. *oxytoca* NifK reduced nitrogenase activity in *E*. *coli* (Yang et al., 2018). The way to solve this problem is to put NifK on the C-terminal of fusion protein.

### Co-expression of nine Nif proteins (NifH, NifB, NifD, NifK, NifE, NifN, NifX, HesA, and NifV) from *Paenibacillus polymyxa* WLY78 in yeast

**Figure 2:**
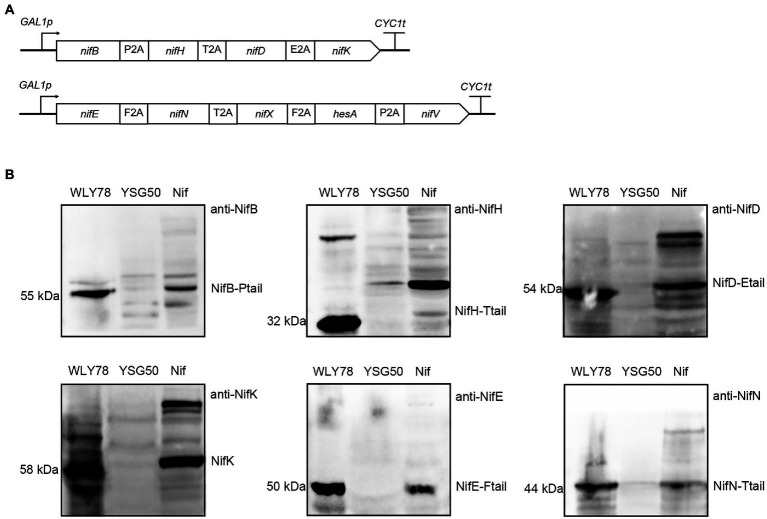
Co-expression of nine Nif proteins in yeast. **(A)** Schematic diagrams of the constructs of nine genes linked *via* different 2A peptides in the recombinant vectors. The four genes (*nifB*, *nifH*, *nifD*, and *nifK*) were linked by P2A, T2A, and E2A peptides, and cloned into pRS423-*GAL1p*. The five genes (*nifE*, *nifN*, *nifX*, *hesA*, and *nifV*) fused by F2A, T2A, F2A, and P2A peptides, and inserted into pRS424-*GAL1p*. All of the *nif* genes were under the control of *GAL1* promoter. **(B)** Western blotting analysis of the recombinant *S*. *cerevisiae* Nif containing nine Nif proteins with anti-NifB, anti-NifH, anti-NifD, anti-NifK, anti-NifE and anti-NifN. *GAL1p* indicates promoter and *CYC1t* indicates terminator. WLY78, *P*. *polymyxa* WLY78 as a positive control; YSG50, *S*. *cerevisiae* YSG50 as a negative control; Nif, *S*. *cerevisiae* Nif.

Our previous studies have revealed that *P*. *polymyxa* WLY78 possesses a minimal *nif* gene cluster composed of nine genes (*nifBHDKENXhesAnifV*). The nine *nif* genes from *P*. *polymyxa* WLY78 enabled *E*. *coli* to synthesize active nitrogenase (Wang et al., 2013). Here, we try to investigate whether the nine *nif* genes from *P*. *polymyxa* WLY78 enabled *S*. *cerevisiae* to synthesize active nitrogenase. The nine genes (*nifBHDKENXhesAnifV*) from *P*. *polymyxa* WLY78, codon optimized for yeast, were synthesized and linked by 2A peptides (P2A, T2A, E2A, and F2A) into two ORFs: (*nifB*-P2A-*nifH*-T2A-*nifD*-E2A-*nifK*) and (*nifE*-F2A-*nifN*-T2A-*nifX*-F2A-*hesA*-P2A-*nifV*) that were under control of *GAL1* promoter (Figure 2A). The recombinant yeast strain carried the nine Nif proteins linked *via* 2A peptide is called *S*. *cerevisiae* Nif.

Total protein extracts were prepared from the recombinant *S*. *cerevisiae* Nif to test expression of nine Nif proteins from two large ORFs. Western blotting with the six antibodies against the proteins NifH, NifD, NifK, NifB, NifE, and NifN of *P*. *polymyxa* WLY78 showed that all six Nif proteins were detectable in the protein extracts and had similar sizes with those of *P*. *polymyxa* WLY78 (Figure 2B). The data suggest that Nif proteins could be correctly co-expressed and cleaved in *S*. *cerevisiae*. The bands of the proteins (NifD, NifK, NifB, NifE and NifN) are stronger that of NifH. We deduce that the band strength of NifH appear weaker than other proteins (eg. NifB) may be result from its low antibody titer. Since protein numbers are too high, it is hard to estimate the cleavage efficiency mediated by the 2A peptides. Co-expression of four genes was applied to investigate reprogramming by using three 2A peptides ((P2A, T2A, and E2A) to allow expression of the four reprogramming factors in a single vector (Carey et al., 2009). The protein expression level from the first to fourth position in a quad-cistronic construct was characterized and the protein expression gradually decreased toward the 3′ end in quad-cistronic constructs (Liu et al., 2017). As we know, 5 proteins linked *via* 2A in this study are the highest number to be co-expressed in yeast. Finally, we measured the nitrogenase activity of the recombinant *S*. *cerevisiae* Nif by using acetylene reduction assays (Wang et al., 2013), but the strain did not show activity. As we know, nitrogenase requires both active NifH and NifDK. Since NifS and NifU for synthesis of Fe-S cluster are required for the active NifH. Thus, we will co-express NifU, NifS and NifH in yeast to check the function of NifH in the follow studies.

### Co-expression of *Paenibacillus polymyxa* NifH and *Klebsiella oxytoca* NifU and NifS in yeast

**Figure 3:**
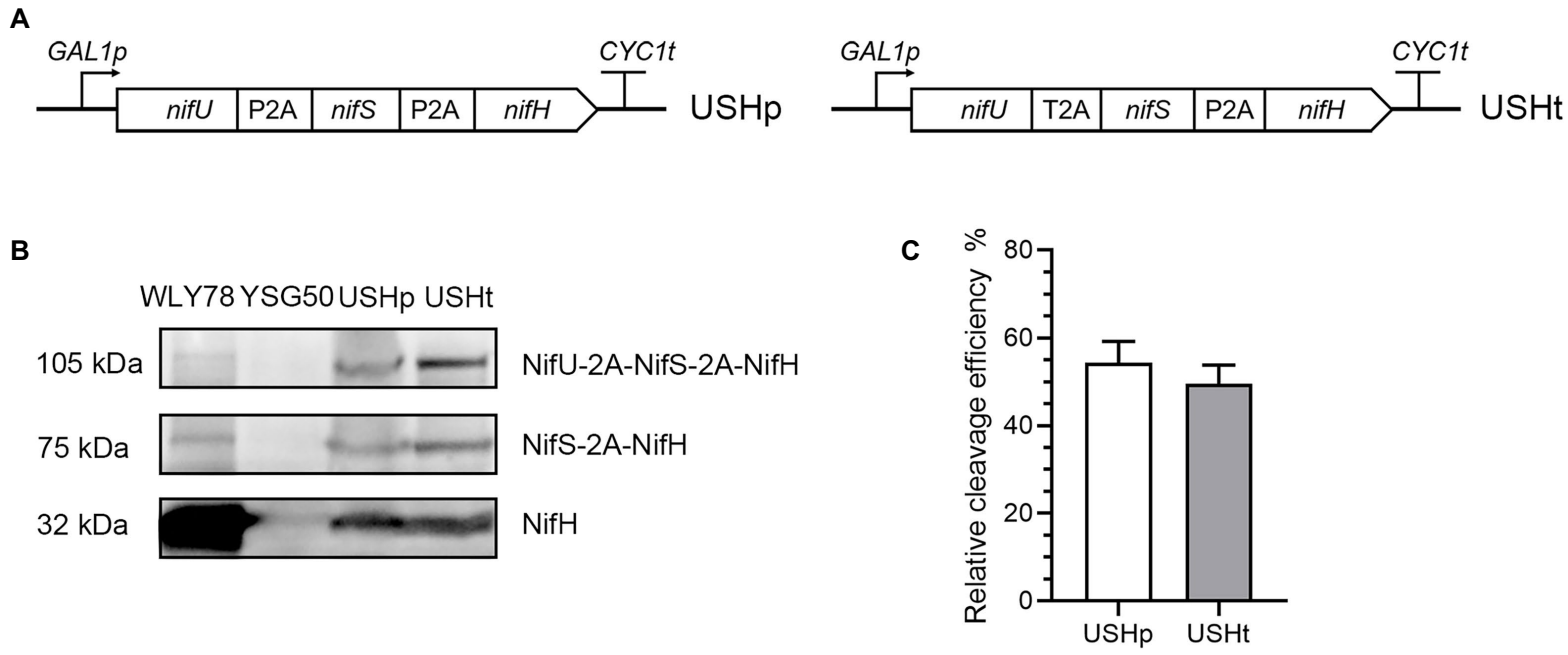
Co-expression of *P*. *polymyxa* NifH and *K*. *oxytoca* NifU and NifS linked *via* 2A peptides in yeast. **(A)** Scheme of *nifU*-2A-*nifS*-2A-*nifH* fusion under control of *GAL1* promoter in the constructed recombinant vectors. **(B)** Western blotting analysis of NifH and its fusions in the recombinant strains *S*. *cerevisiae* USHp and USHtm. *S*. *cerevisiae* YSG50 was used as negative control and *P*. *polymyxa* WLY78 was used as positive control. **(C)** Quantitation of cleavage efficiency of the second 2A sequence. Cleavage efficiency = NifH/(NifH+NifS-2A-NifH+NifU-2A-NifS-2A-NifH) *100%. The band intensity on the Western blotting measured by ImageJ software. Error bars indicated SEM, *n* = 3. USHp: *S*. *cerevisiae* USHp, USHt: *S*. *cerevisiae* USHt.

Function of NifH (Fe protein) is conferred by Fe_4_-S_4_ cluster that bridges the NifH subunits in the Fe protein homodimer (Hoffman et al., 2014). NifU and NifS are responsible for synthesis of Fe-S cluster. Most of N_2_-fixing bacteria, such as *Klebsiella oxytoca*, have *nifS* and *nifU* genes encoding NifU and NifS, but *P*. *polymyxa* WLY78 lacks *nifU* and *nifS* genes. Also, our previous studies have shown that the *K*. *oxytoca nifU* and *nifS* could enhanced the nitrogenase activity of the recombinant *E*. *coli* strain that carried nine *nif* genes (*nifBHDKENXhesAnifV*) from *P*. *polymyxa* WLY78. Here, *K*. *oxytoca nifU*, *nifS* and *P*. *polymyxa nifH* whose 5′-coding region was tagged by 10 histidines, codon optimized for yeast, were fused into a single ORF (*nifU*-P2A-*nifS*-P2A-*nifH*-10 × His) *via* two P2A peptides or a single ORF (*nifU*-T2A-*nifS*-P2A-*nifH*-10 × His) *via* a T2A peptide and a P2A peptide. Each of the two ORFs was cloned into pRS423-*GAL1p* vector to be expressed under the control of *GAL1* promoter and then these recombinant plasmids were transformed into *S*. *cerevisiae* YSG50, respectively. Thus, strains *S*. *cerevisiae* USHp (carrying *nifU*-P2A-*nifS*-P2A-*nifH*-10 × His) and *S*. *cerevisiae* USHt (carrying *nifU*-T2A-*nifS*-P2A-*nifH*-10 × His) were generated (Figure 3A).

To investigate whether NifH was expressed in yeast, total protein extracts were prepared from yeast and analyzed by Western blotting with anti-NifH. The cleaved form NifH (32 kDa) and the uncleaved forms NifS-2A-NifH (75 kDa) and NifU-2A-NifS-2A-NifH (105 kDa) were detectable in recombinant *S*. *cerevisiae* USHp and *S*. *cerevisiae* USHt, while they were not detected in the recipient *S*. *cerevisiae* YSG50 (Figure 3B). The cleaved NifH (32 kDa) was the major form in yeast and it had the similar molecular size with that of *P*. *polymyxa* NifH.

As shown in Figure 3C, the cleavage efficiency of the P2A in *S*. *cerevisiae* USHp and in *S*. *cerevisiae* USHt was similar at 49–52%, which is lower than the 80% cleavage efficiency in the NifB-P2A-NifH where NifB and NifH were linked by P2A. Our data are consistent with the reports that the highest protein expression was found at the first position in tri-cistronic constructs (Liu et al., 2017). It is also found in co-expression of cytochrome P450cc, adrenodoxin and adrenodoxin linked by two 2A peptides, the cleavage efficiency of the first 2A ranged 90–96%, while the cleavage efficiency of the second 2A peptides ranged from 11 to 68% (Efimova et al., 2021).

### Yeast NifH expressed from the huge protein (NifS-2A-NifU-2A-NifH) exhibits activity of Fe protein

**Figure 4:**
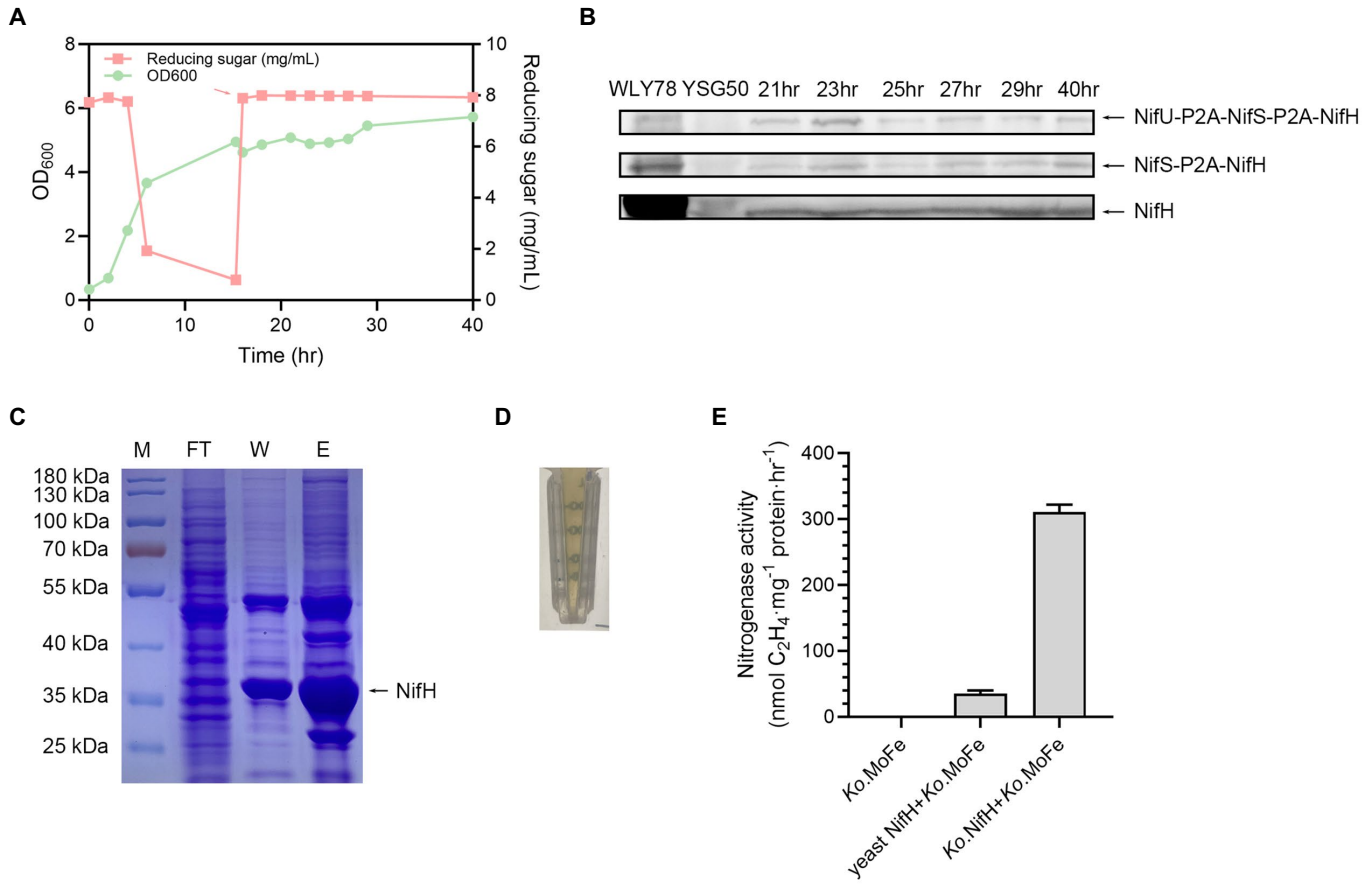
Growth of *S*. *cerevisiae* USHp and detection of expression and activity of NifH. **(A)** Batch fermentation process of *S*. *cerevisiae* USHp. OD_600_ value (green) and concentration of deoxidize sugar (red) were continuously monitored during the process. The red arrow indicates addition time of 2% galactose and trace-metal solution. During the fermentation process, pH was automatically controlled at 5.8 by 5 M KOH, and temperature was controlled at 30°C. **(B)** Western blotting analysis of NifH expression with anti-NifH during the fermentation process. NifH was cleaved form, while NifS-P2A-NifH and NifU-P2A-NifS-P2A-NifH were fusion protiens. WLY78, *P*. *polymyxa* WLY78 as a positive control; YSG50, *S*. *cerevisiae* YSG50 as a negative control. **(C)** Purification of NifH proteins. Ni^2+^ affinity column was used for the purification of the His-tagged NifH. M, molecular weight marker; FT, flow-through fractions; W, protein fractions eluted after washing with washing buffer; E, protein fractions eluted after washing with elution buffer. Arrow point to purified NifH protein. **(D)** The characteristic brown color of nitrogenase Fe protein. **(E)** Activity assays of yeast NifH protein. Fe protein activity was analyzed by assessing the nitrogenase activity (acetylene reduction activity) of the mixtures of NifH protein and the *K*. *oxytoca* MoFe protein (*Ko*. MoFe). *Ko*. MoFe without *K*. *oxytoca* Fe protein (*Ko*. Fe) as a negative control. *Ko*. Fe with addition of *Ko*. MoFe as a positive control. The error bars indicated SEM, *n* = 3.

*Saccharomyces cerevisiae* USHp cells were initially grown in medium containing 0.6% glucose with strong aeration for about 15 h. After glucose was nearly consumed, 2% galactose was added to culture to induce expression of *nif* genes and also nitrogen gas was used to induce anerobic fermentation for more than 20 h (Figure 4A). Western blotting analysis showed that NifH was produced at 21, 25, 27, 29, and 40 h during the fermentation process (Figure 4B). The cleaved NifH was the major form, and the un-cleaved NifH forms also existed.

His-tagged NifH was purified from *S*. *cerevisiae* USHp cells after 40 h of cultivation by using anaerobic Ni^2+^ affinity chromatography inside a glove box (Figure 4C). The purified NifH exhibited brown color which was the characteristic of nitrogenase Fe protein (Figure 4D). The Fe protein activity of the purified yeast NifH was analyzed by assaying nitrogenase activity in the mixture of yeast NifH and pure *K*. *oxytoca* MoFe protein. In absence of yeast NifH, pure *K*. *oxytoca* MoFe protein did not exhibit nitrogenase. As observed in positive control of mixtures of pure *K*. *oxytoca* Fe and FeMo proteins, yeast NifH showed nitrogenase activity in presence of *K*. *oxytoca* MoFe protein. The data indicate that the expressed *P*. *polymyxa* NifH in yeast was capable of donating electrons to MoFe protein from *K*. *oxytoca* (Figure 4E). The expressed *P*. *polymyxa* NifH in yeast did not require NifM to render active, consistent with the finding that there is no *nifM* gene in the genome of *P*. *polymyxa*. In contrast, *A*. *vinelandii* NifH requires NifM to render active Fe protein (López-Torrejón et al., 2016). However, the activity of the purified yeast NifH is 5–10% of that of *K*. *oxytoca* Fe protein. We deduce that the low activity of the purified yeast NifH results from two reasons. One reason is that *K*. *oxytoca* MoFe protein does not match *P*. *polymyxa* Fe protein (NifH) very well. The other reason is that the nitrogenase activity in the mixture of *K*. *oxytoca* Fe and MoFe protein is much higher than that in the mixture of *P*. *polymyxa* Fe and MoFe. Recently, we have revealed that the nitrogenase activity in the mixture of Fe and MoFe purified from *Paenibacillus sabinae* T27 is much higher than in the mixture of the purified *P*. *sabinae* MoFe and *K*. *oxytoca* Fe or *A*. *vinelandii* Fe. Also, the nitrogenase activity in the mixture of *K*. *oxytoca* Fe and MoFe protein is much higher than that in the mixture of *P*. *sabinae* Fe and MoFe (Li et al., 2021a). Recently, we have revealed that the *sufCDSUB* operon, *nifS-like* and *yutI* genes were involved in the Fe–S cluster biosynthesis of nitrogenase in *P*. *polymyxa* WLY78 (Li et al., 2021b). In the future, we can co-express *nifH* and these genes involved in Fe–S cluster biosynthesis of *P*. *polymyxa* WLY78 in yeast to investigate whether the Fe activity can be enhanced.

In summary, we use 2A peptides to co-express multiple proteins involved in synthesis of the complex nitrogenase system. Our results for the first time demonstrate that Nif proteins linked *via* 2A peptides can be successfully co-expressed in *S*. *cerevisiae*. All the four 2A peptides (P2A, T2A, E2A and F2A) evaluated show cleavage with different efficiency, ranging from ~50% to ~90%. The presence of a 2A tail in NifH, NifB and NifD dose not affect the activity of these Nif proteins, but 2A tail causes a complete loss of NifK activity. Nine Nif proteins (NifH, NifB, NifD, NifK, NifE, NifN, NifX, HesA and NifV) from *P*. *polymyxa* WLY78 are successfully co-expressed in yeast. Yeast NifH co-expressed from *P*. *polymyxa* NifH and *K*. *oxytoca* NifS and NifU exhibits activity of Fe protein.

## Materials and methods

### Strains and media

Bacterial strains and yeast strains used in this study are shown in Supplementary Table S1. *S*. *cerevisiae* YSG50 was used as host for *nif* gene expression. *S*. *cerevisiae* Y2H Gold was used as a host in Yeast two hybrid system for detecting the cleavage efficiency of 2A linkers *in vivo*. *S*. *cerevisiae* YSG50 and Y2H Gold strains were usually grown at 30°C in YPD medium (20 g/l tryptone, 10 g/l yeast extract, 20 g/l glucose). The yeast transformants were screened at 30°C in SC medium without tryptophan (SC-Trp), or without tryptophan, histidine and adenine (SC-Trp-His-Ade). *E*. *coli* DH5α was used to routine cloning and plasmid propagation. Luria-Bertani broth. *P*. *polymyxa* WLY78 was grown in nitrogen-limited medium (Wang et al., 2013). When appropriate, antibiotics were added at the following concentrations: 100 μg/mL Ampicillin and 5 μg/mL Erythromycin for maintenance of plasmids in bacterial strains. Primers and plasmids used in this study were listed in Supplementary Tables S3, S4, respectively.

### The codon-optimized *nif* gene sequences

The nine genes (*nifBHDKENXVhesA*) from *P*. *polymyxa* WLY78, codon-optimized for yeast, were synthesized together with 2A peptides (P2A, T2A, E2A, and F2A) and they were separated to two large ORFs (GeneScript Biotech Co., China) (Supplementary Table S2). Namely, *nifB*-P2A-*nifH*-T2A-*nifD*-E2A-*nifK* was synthesized as an ORF that was cloned to vector pUC57, generating the recombinant plasmid pUCE-c1, and *nifE*-F2A-*nifN*-T2A-*nifX*-F2A-*hesA*-P2A-*nifV* was synthesized as an ORF that was cloned to vector pUC57, generating the recombinant plasmid pUCE-c2. The *nifS* and *nifU* from *K*. *oxytoca* were codon-optimized for yeast (Liu et al., 2019).

### Construction of plasmids pRS423-c1 and pRS424-c2 for expressing nine *nif* genes from *Paenibacillus polymyxa* WLY78 in yeast

Plasmid pRS423-c1 containing *nifB-P2A-nifH-T2A-nifD-E2A-nifK* fusion was constructed by PCR amplifying a 5,550 bp fragment containing *nifB-P2A-nifH-T2A-nifD-E2A-nifK* fusion from pUCE-c1 with primers f34/r34 and by ligating the PCR fragment to EcoRI/SalI digested vector pRS423-*GAL1p* with T4 ligase, pRS424-c2 plasmid containing *nifE-F2A-nifN-T2A-nifX-F2A-hesA-P2A-nifV* fusion was constructed by PCR amplifying a 5,235 bp fragment containing *nifE-F2A-nifN-T2A-nifX-F2A-hesA-P2A-nifV* fusion from pUCE-c2 with primers f35/r35 and by ligating the PCR fragment to EcoRI/SalI digensted vector pRS423-*GAL1p* with T4 ligase. The two plasmids were co-transformed into *S*. *cerevisiae* YSG50, and the transformants were selected on SC-His and identified by PCR analysis. A positive yeast transformant carrying 9 *nif* genes (*nifBHDKENX hesA nifV*) was designed as *S*. *cerevisiae* Nif (Supplementary Table S1).

### Construction of plasmids pRS423-USHp and pRS423-USHt for co-expression of *Klebsiella oxytoca* NifU and NifS and *Paenibacillus polymyxa nifH* in yeast

Plasmid pRS423-USHp carrying *nifU-P2A-nifS-P2A-nifH-His* tag was constructed as follows. An 837 bp fragment (*nifU-P2A*) was PCR amplified from pUCE-U with primers f36/r36, of which primer r36 contained the *nifU’*-end (without stop codon) and partial P2A sequences. A 1200 bp fragment (*P2A-nifS-P2A*) was PCR amplified was PCR amplified from pUCE-S with primers f37/r37, of which primer f37 contained partial P2A sequences at its 5′-end and primer r37 contained the *nifS’*-end (without stop codon) and partial P2A sequences. A 867 bp fragment (*P2A-nifH*) was PCR amplified from pUCE-c1 with primers f38/r38, of which primer f38 contained partial P2A sequences and primer r38 contained the 3′-end of *nifH’* containing stop codon. A fragment (*nifU-P2A-nifS-P2A*) was obtained by fusing *nifU-P2A* and *P2A-nifS-P2A* using overlap extension PCR with primers f36/r37. The *nifU-P2A-nifS-P2A* fragment was fused to *P2A-nifH* by using overlap extension PCR with primers f36/r38, generating a 3,018 bp fragment (*nifU-P2A-nifS-P2A-nifH*). Finally, the fragment (*nifU-P2A-nifS-P2A-nifH*) was assembled to vector pRS423-*GAL1p* digested with EcoRI and SalI, generating plasmid pRS423-USH1. Finally, the whole sequences of plasmid pRS423-USH1 was PCR amplified with primers f39/r39 that contained 10xHis sequences (His tag), generating a 9,501 bp fragment (His *tag*-pRS423-*nifU-P2A-nifS-P2A-nifH-His tag*). The linear PCR fragment was circled by assembly enzyme, generating plasmid pRS423-USHp.

pRS423-USHt carrying *nifU-T2A-nifS-P2A-nifH-His tag* was constructed as follows. An 837 bp fragment (*nifU-T2A*) was PCR amplified from pUCE-U with primers f36/r40, of which primer r40 contained the 3′-end of *nifU* without stop codon and partial T2A. A 1200 bp fragment (*T2A-nifS-P2A*) was amplified from pUCE-S with primers f41/r37, of which f41 contained 5′-end of *nifS* and partial T2A sequences and primer r37 contained 3′-end of *nifS* without stop codon and partial T2A sequences. A 867 bp fragment (*P2A-nifH*) was PCR amplified with primers f38/r38, of which primer f38 contained partial P2A sequences and primer r38 contained 3′-end of *nifH* with a stop codon. The *nifU-T2A-nifS-P2A* was obtained by fusing *nifU-T2A* and *T2A-nifS-P2A* using overlap extension PCR with primers f36/r37. A 3015 bp *nifU-T2A-nifS-P2A-nifH* fragment was obtained by fusing *nifU-T2A-nifS-P2A* and *P2A-nifH* fragments with primers f36/r38. The fragment was assembled to vector pRS423-*GAL1p* digested by EcoRI and SalI, generating plasmid pRS423-USH2. The whole sequences of plasmid pRS423-USH2 was PCR amplified with primers f39/r39 that contained 10xHis sequences (HiS tag), generating a 9,489 bp fragment (*His tag-pRS423-nifU-T2A-nifS-P2A-nifH-His tag*). The fragment was circled by assembly enzyme *via* homologous recombination, yielding plasmid pRS423-USHt.

Plasmids pRS423-USHp and pRS423-USHt were individually transformed into *S*. *cerevisiae* YSG50 and transformants were selected on SC-His and identified by PCR analysis. Two positive transformants were *S*. *cerevisiae* USHp carrying plasmid pRS423-USHp and *S*. *cerevisiae* USHt carrying pRS423-USHt (Supplementary Table S1).

### Construction of *Paenibacillus polymyxa nif* mutants and other recombinant plasmids

Plasmids for assaying cleavage efficiency of of NifB and NifH fusion protein linked *via* different 2A peptides in yeast and for yeast two hybrid (Y2H) assay were shown in Supplementary Material. Construction of Δ*nifB*, Δ*nifH*, Δ*nifD*, and Δ*nifK* mutants of *P*. *polymyxa* WLY78 and their complement strains were shown in Supplementary Material.

### Yeast two-hybrid assay

*Saccharomyces cerevisiae* Y2H Gold was a host used in yeast two hybrid. The derivatives of *S*. *cerevisiae* Y2H Gold carrying the Nif polyprotein linked *via* 2A peptide were grown on SC medium lacking tryptophan (SC-Trp) and on SC-Trp-His-Ade agar plates.

### Tailing-tolerance assay

To determine the tolerance of Nif protein to the C-terminal tail, *nif* gene carrying 2A peptide sequence (tail) was used to complement the *nif* deletion mutant of *P*. *polymyxa* WLY78, generating complement strains. Nitrogenase activities of wild-type, mutants and complement strains were comparatively determined.

### Western blotting

For immunoblot detection of Nif protein expression and 2A self-cleavage efficiency, *S*. *cerevisiae* YSG50 (wild-type) and derivatives were grown in 50 ml flasks containing 20 ml of SD medium until glucose was consumed. Then, 2% galactose was added to induce *nif* genes separated by 2A sequences expression. Cultures were collected immediately after 9 h of incubation with shaking at 30°C and 200 rpm. Cells of *P*. *polymyxa* WLY78 cultivated anaerobically in nitrogen-limited medium were collected and then used as a positive control. The cells pellets were suspended in 200 μL 1 × SDS loading buffer and boiled for 10 min, and then, 20 μL of each sample were loaded onto 12% separating gels. Subsequently, proteins on the gels were transferred to NC membranes (Huaxingbio, China). The antibodies (anti-NifH, anti-NifD, anti-NifK, anti-NifB, anti-NifE, and anti-NifN) were used at a dilution of 1: 10000. The secondary antibody goat anti-rabbit IgG-HRP (Huaxingbio, China) was also used at 1: 10000. Immunochemiluminescene was done by using the NcmECL Western Blot Kit (NCM Biotech, China). The band intensity on the Western blots was analyzed using ImageJ. The cleavage efficiency of 2A linkers was calculated as (cleaved form) / (cleaved form + uncleaved form) × 100%.

### Growth of yeast strains and purification of yeast NifH

*Saccharomyces cerevisiae* USHp was used for purification of NifH protein. It was cultivated in 500 ml of SD medium with shaking at 200 rpm at 30°C as described by Liu et al. (2019). The cells were lysed in a high pressure homogenizer (PhD Technology International LLC, United States) at 25,000 lb. per square inch. His-tagged NifH was purified by Ni^2+^ affinity chromatography (QIAGEN, Germany) under anaerobic conditions inside a glovebox (M. Braun Inertgas systems, Germany). Purified NifH protein was stored in liquid N_2_.

### Determination of Fe protein activity

Fe protein activity of the yeast NifH was analyzed by assaying nitrogenase activity *in vitro*. Yeast NifH protein was mixed with pure *K*. *oxytoca* MoFe protein, and ATP-regeneration mixture 5 mM ATP, 40 mM creatine phosphate, 10 mM MgCl_2_, 20 mM sodium dithionite, 0.125 mg/mL creatine phosphokinase, 40 mM MOPS-KOH (pH 7.4) (Li and Burris, 1983). Positive control reactions were carried out with pure Fe protein and MoFe protein from *K*. *oxytoca*.

### Nitrogenase activity assay

Nitrogenase activities of *P*. *polymyxa* WLY78 and its derivatives were determined by acetylene reduction assay according to method described by Wang et al. (2018).

## Data availability statement

The raw data supporting the conclusions of this article will be made available by the authors, without undue reservation.

## Author contributions

SC designed research. MW carried out gene clone and assembly, protein purification, and biochemical studies. XL contributed to gene clone. YS contributed to fermentations. MW and SC analyzed data and wrote the manuscript. All authors contributed to the article and approved the submitted version.

## Funding

This work was supported by the National Key Research and Development Program of China Award (No. 2019YFA0904700).

## Conflict of interest

The authors declare that the research was conducted in the absence of any commercial or financial relationships that could be construed as a potential conflict of interest.

## Publisher’s note

All claims expressed in this article are solely those of the authors and do not necessarily represent those of their affiliated organizations, or those of the publisher, the editors and the reviewers. Any product that may be evaluated in this article, or claim that may be made by its manufacturer, is not guaranteed or endorsed by the publisher.
