# Supplementary material for: Assembly of nitrogenase biosynthetic pathway in Saccharomyces cerevisiae by using polyprotein strategy
Source: Front Microbiol. 2023 Mar 2;14:1137355. doi: 10.3389/fmicb.2023.1137355 (PMC10017450; doi:10.3389/fmicb.2023.1137355)
Supplement: Supplementary file 1 [file Data_Sheet_1.docx]

**Supporting information**

Supplementary materials and methods

Supplementary Figures 1-2

Supplementary Tables 1-4

Supplementary references

**Construction of plasmids for assaying cleavage efficiency of of NifB and NifH fusion protein linked via four different 2A peptides in yeast**

To determine the cleavage of NifB and NifH linked via different 2A peptides, 5 recombinant plasmids were constructed. (1) pRS423-BH plasmid carrying *nifB-nifH* fusion. Here, a 1497 bp *nifB* gene was PCR amplified from pUCE-c1 with f1/r1 and an 867 bp *nifH* gene was PCR amplified from pUCE-c1 with f2/r2, of which primer r2 carried a stop codon at its 3’-end. A 2364 bp *nifB-nifH* fragment was generated by fusing *nifB* and *nifH* fragments using overlap extension PCR with primers f1 and r2. Then, the *nifB-nifH* fragment without 2A peptide linker was inserted between *GAL1* promoter (*GAL1p*) and *CYC* terminator (*CYC1t*) in vector pRS423-*GAL1p* digested by EcoRI and SalI, yielding plasmid pRS423-BH that was used as a negative control. (2) pRS423-BP2AH plasmid carrying *nifB-P2A-nifH*. A 2421 bp *nifB-P2A-nifH* fragment was PCR amplified from pUCE-c1 with primers f1/r2, of which primer r2 contained a stop codon. The *nifB-P2A-nifH* fragment was inserted between *GAL1p* and *CYC1t* in vector pRS423-*GAL1p* digested by EcoRI and SalI, yielding plasmid pRS423-BP2AH. (3) pRS423-BT2AH plasmid carrying *nifB-T2A-nifB*. A *nifB-T2A* fragment was PCR amplified from pUCE-c1 with primers f1/r4, of which primer r4 contained T2A sequences at its 5’-end, and a *T2A-nifH* fragment was PCR amplified from pUCE-c1 with primers f5/r2, of which primer f5 contained T2A sequence at its 3’-end. Then, a 2418 bp *nifB-T2A-nifH* fragment was generated by fusing *nifB-T2A* and *T2A-nifH* fragments using overlap extension PCR with primers f1/r2. The *nifB-T2A-nifH* fragment was inserted between *GAL1p* and *CYC1t* in vector pRS423-*GAL1p* digested by EcoRI and SalI, yielding plasmid pRS423-BT2AH. (4) pRS423-BE2AH plasmid carrying *nifB-E2A-nifH*. A *nifB-E2A* fragment was PCR amplified from pUCE-c1 with primers f1/r6, of which r6 contained E2A sequences at its 5’-end. An *E2A-nifH* fragment was PCR amplified from pUCE-c1 with primers f7/r2, of which primer f7 contained E2A sequences at its 3’-end. A 2424 bp *nifB-E2A-nifH* fragment was generated by fusing *nifB-E2A* and *E2A-nifH* fragments using overlap extension PCR with primers f1/r2. The *nifB-E2A-nifH* fragment was inserted between *GAL1p* and *CYC1t* in vector pRS423-*GAL1p* digested by EcoRI and SalI, yielding plasmid pRS423-BE2AH. (5) pRS423-BF2AH plasmid carrying *nifB-F2A-nifH*. A *nifB-F2A* fragment was PCR amplified from pUCE-c1 with primers f1/r8, of which primer r8 contained F2A sequences at its 5’-end. A *F2A-nifH* fragment was PCR amplified from pUCE-c1with primers f9/r2, of which primer f9 contained F2A sequences at its 5’-end. A 2430 bp *nifB-F2A-nifH* fragment was generated by fusing *nifB-F2A* and *F2A-nifH* using overlap extension PCR with primers f1/r2. The *nifB-F2A-nifH* fragment was inserted between *GAL1p* and *CYC1t* in vector pRS423-*GAL1p* digested by EcoRI and SalI, yielding plasmid pRS423-BF2AH. The 5 plasmids were individually transformed to *S. cerevisiae* YSG50, and 5 positive transformants were obtained. These yeast strains included *S. cerevisiae* BH, *S. cerevisiae* P2A, *S. cerevisiae* T2A, *S. cerevisiae* E2A and *S. cerevisiae* F2A (Table S1).

**Construction of plasmids for yeast two hybrid (Y2H) assay**

Yeast two hybrid was used to determine whether NifB and NifH linked via 4 different 2A peptides were cleaved. Vector pGBKT7 (TaKaRa Co. China) carries a *GAL4* DNA-Binding domain (BD) that is under control of *ADH1* promoter (*ADH1p*) and has a ADH terminator (*ADH1t*). Vector pGADT7 (TaKaRa Co. China) carries a *GAL4* activation domain (AD) that is under the control of *ADH1* promoter and has a *ADH1* terminator. pGBKT7-AD, a middle vector, was constructed. For doing this, a 345 bp *GAL4* active domain (AD) was PCR amplified from vector pGADT7 with primers f10/r10, of which primer r10 carried a top codon at its 3’-end. Then, the *GAL4* activation domain (AD) was assembled to the upstream of *ADH1* terminator in vector pGBKT7 digested by EcoRI and BamHI, generating plasmid pGBKT7-AD containing both AD and BD domains of *GAL4*.

Then, five DNA fragments, including a NifB and NifH fusion without 2A peptide, and four NifB and NIfH fusions linked via 4 different 2A peptides, were PCR amplified with primers f11/r11 from 5 different plasmids constructed above. Namely, a 2361 bp *nifB-nifH* fragment was PCR amplified from plasmid pRS423-BH with primers f11/r11. A 2418 bp *nifB-P2A-nifH* fragment from plasmid pRS423-BP2AH and a 2418 bp *nifB-T2A-nifH* fragment from plasmid pRS423-BT2AH were PCR amplified with primers f11/r11. A 2424 bp *nifB-E2A-nifH* fragment from plasmid pRS423-BE2AH and a 2430 bp *nifB-F2A-nifH* fragment from plasmid pRS423-BF2AH were PCR amplified with primers f11/r11. Then, each of the five NifB-NifH fusions with or without 2A peptide was individually inserted between BD and AD domains of pGBKT7-AD vector digested by EcoRI, with NifB in frame being fused to BD domain and with NifH in frame being fused to AD domain, and then five recombinant plasmids pGBKT7-BH-AD, pGBKT7-BP2AH-AD, pGBKT7-BT2AH-AD, pGBKT7-BE2AH-AD and pGBKT7-BF2AH-AD were produced (Table S4). Each of the five plasmids was transformed to *S. cerevisiae* Y2H Gold and then five positive transformants were selected on SC medium lacking tryptophan (SC-Trp). The five positive transformants were *S. cerevisiae* Y2H-BH, *S. cerevisiae* Y2H-BP2AH, *S. cerevisiae* Y2H-BT2AH, *S. cerevisiae* Y2H-BE2AH and *S. cerevisiae* Y2H-BF2AH. *S., cerevisiae* Y2H-BD carrying an empty vector pGBKT7 was produced by transforming pGBKT7 to *S. cerevisiae* Y2H Gold.

**Construction** **of** **Δ*nifB*, Δ*nifH,* Δ*nifD* and Δ*nifK* mutants of *P. polymyxa* WLY78**

The in-frame-deletion mutants Δ*nifH*, Δ*nifB,* Δ*nifD* and Δ*nifK* were constructed via homologous recombination using the suicide plasmid pRN5101 as described previously (Wang et al., 2018). The upstream and downstream fragments flanking the coding region of *nifH*, *nifB, nifD* and *nifK* were PCR amplified from the genomic DNA of *P. polymyxa* WLY78, respectively. The primers (f12/r12, f13/r13, f20/r20, f21/r21; f24/r24, f25/r25; f29/r29, f30/r30) used for these PCR amplifications were listed in Table S3. The upstream and downstream fragments of four genes were then fused to *Bam*HI and *Hind*III-digested vector pRN5101 by using Gibson assembly master mix (New England Biolabs), generating four recombinant plasmids, pRN5101-ΔH, pRN5101-ΔB, pRN5101-ΔD and pRN5101-ΔK. Then, each of these recombinant plasmids was transformed into *P. polymyxa* WLY78, and the single-crossover transformants were screened for erythromycin resistance (Em^r^). Subsequently, marker free deletion mutants (the double-crossover transformants) were selected from the initial Em^r^ transformants after several rounds of nonselective growth at 39°C. The marker-free deletion mutants were confirmed by PCR amplification and DNA sequencing analysis.

**Complementation of Δ*nifH* mutant of *P. polymyxa* WLY78**

For complementation of Δ*nifH* mutant of *P. polymyxa* WLY78, pRN5101-proH plasmid containing *Pnif-nifH* was constructed. A 310 bp promoter region (*Pnif*) of *nifB* and a 867bp *nifH* gene were PCR amplified with primers f14/r14 and primers f15/r15 from *P. polymyxa* WLY78, respectively. Then, a 1177 bp *Pnif-nifH* fragment was obtained by overlap extension PCR with primers f14/r15. Finally, *Pnif-nifH* fragment was assembled to vector pRN5101 digested by BamHI and HindIII, generating plasmid pRN5101-proH that contained *Pnif-nifH*.

Here, plasmids carrying *nifH*-2A tails were constructed for complementing Δ*nifH* mutant of *P. polymyxa* WLY78**.** The whole sequences of plasmid pRN5101-proH was PCR amplified with primers f16/r16 containing P2A sequences (here called Ptail) that were added in front of stop codon of *nifH*, generating a 10159 bp fragment (*Ptail-pRN5101-Pnif-nifH-Ptail*). Then, the 10159 bp linear fragment was circled by using assembly enzyme via homologous recombination, generating plasmid pRN5101-HPtail that contained *Pnif-nifH*-*Ptail*. Similarly, a 10159 bp fragment (*Ttail-pRN5101-**Pnif-nifH-Ttail*) was PCR amplified from pRN5101-proH by using primers f17/r17 containing T2A sequences (called Ttail) and then it was circled by assembly enzyme, generating plasmid pRN5101-HTtail that contained *Pnif*-*nifH*-*Ttail***.** A 10159 bp fragment (*Etail-pRN5101-Pnif-nifH-Etail*) was PCR amplified with primers f18/r18 containing E2A sequences (called Etail) and then it was circled by using assembly enzyme, generating plasmid pRN5101-Etail that contained *Pnif-nifH-Etail*. A 10160 bp fragment (*Ftail-pRN5101-Pnif-nifH-Ftail*) was PCR amplified with primers f19/r19 containing F2A sequences (called Ftail) and then it circled by assembly enzyme, generating plasmid pRN5101-Ftail that contained *Pnif-nifH-Ftail*. Each of these plasmids was transformed to Δ*nifH* mutant of *P. polymyxa* WLY78 and positive transformants were selected and identified by PCR.

**Complement of Δ*nifB*** **mutant of *P. polymyxa* WLY78**

pRN5101-proB plasmid containing *Pnif-nifB*, was constructed as follows. A 1810 bp fragment (*Pnif-nifB*) containing *nifB* promoter and coding region of *nifB* was PCR amplified from *P. polymyxa* WLY78 with primers f14/r22 and then it was assembled to vector pRN5101 digested by BamHI and HindIII, generating plasmid pRN5101-proB.

Plasmid pRN5101-Btail containing *nifB-*2A tail was constructed. A 10792 bp fragment (*Ptail-pRN5101-Pnif-nifB-Ptail*) *w*as PCR amplified with primers f23/r23, of which P2A sequences (here called Ptail) was added in front of stop codon of *nifB*. Then, the large fragment was circled by using assembly enzyme, generating plasmid pRN5101-Btail that contained *Pnif-nifB-Ptail.* These plasmids were individually transformed to Δ*nifB* mutant of *P. polymyxa* WLY78 for complementation.

**Complementation of** ***nifD* mutant of *P. polymyxa* WLY78**

Plasmid pRN5101-proD containing *Pnif-nifD* for complementation was constructed. A 300 bp promoter region (called *Pnif*) of *nifB* and a 1449 bp *nifD* gene was PCR amplified from *P. polymyxa* WLY78 with primers f14/r26 and f27/r27, respectively. A 1759 bp *Pnif-nifD* fragment was produced by fusing P*nif* and *nifD* fragments using overlap extension PCR with primers f14/r27. Finally, the *Pnif-nifD* fragment was assembled to BamHI and HindIII digested vector pRN5101, generating plasmid pRN5101-proD.

Plasmid pRN5101-Dtail carrying *nifD-*2A tail was constructed. A 10741bp fragment (*Ptail-pRN5101-Pnif-nifD-Ptail*) was PCR amplified from pRN5101-proD with primers f28/r28 that contained P2A sequences (Ptail) added in front of stop codon of *nifD*. Then, the linear PCR fragment was circled by using assembly enzyme via homologous recombination, producing plasmid pRN5101-Dtail with *Pnif-nifD-Ptail*. These plasmids were individually transformed to Δ*nifD* mutant of *P. polymyxa* WLY78 for complementation.

**Complementation of** **Δ*nifK* mutant of *P. polymyxa* WLY78**

Plasmid pRN5101-proK containing *Pnif-nifK* for complementation was constructed. A 310 bp promoter region (*Pnif*) of *nifB* were PCR amplified with primers f14/r31 and a 1572 bp *nifK* gene was PCR amplified with primers f32/r32 from *P. polymyxa* WLY78. A 1882 bp (*Pnif-nifK*) was produced by overlap extension PCR with primers f14/r32. The *Pnif-nifK* fragment was assembled to vector pRN5101 digested by BamHI and HindIII, generating plasmid pRN5101-proK.

Plasmid pRN5101-Ktail containing *nifK*-2A tail was constructed. The whole length of plasmid pRN5101-proK was PCR amplified with primers f33/r33 that contained P2A sequences added in front of stop of *nifK*, generating a 10865 bp fragment (Ptail-pRN5101*-Pnif-nifK-*Ptail). The linear PCR fragment was circled by assembly enzyme via homologous recombination, generating plasmid pRN5101-Ktail that contained *Pnif-nifK-*Ptail. These plasmids were individually transformed to Δ*nifK* mutant of *P. polymyxa* WLY78 for complementation.


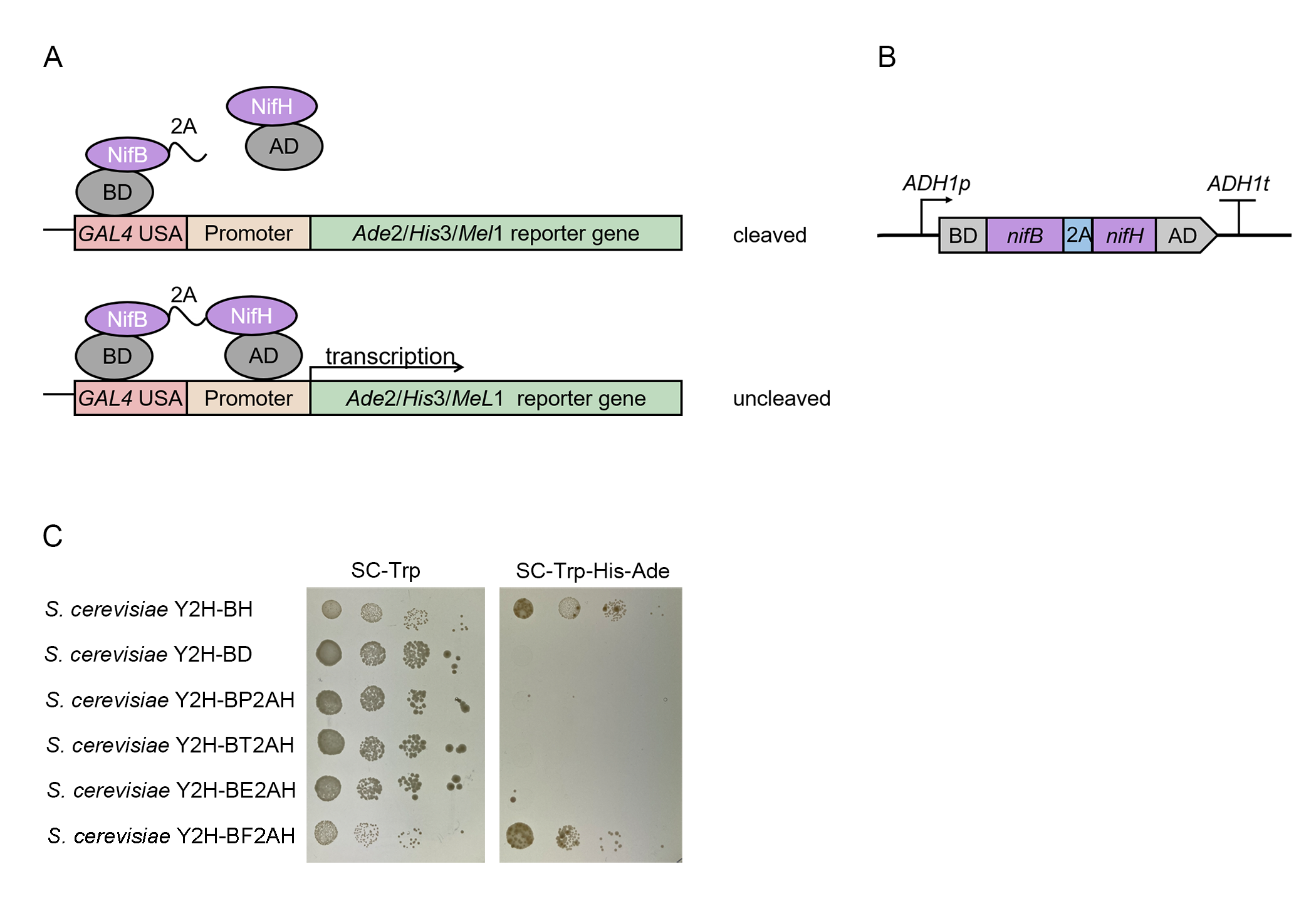


**Figure** **S1**. Yeast two hybrid (Y2H) assay for determination of cleavage efficiency of NifB and NifH linked via four different 2A peptides. (A) Schematic diagrams of model of *GAL4* BD-NifB and *GAL4* AD-NifH linked via 2A peptide, (B) Schematic diagrams of *GAL*4 BD-NifB-2A-NifH-*GAL4* AD as an ORF with *ADH1* promoter at its 5’-end and with *ADH1* terminator at its 3’-end in the constructed recombinant vectors. (C) Y2H assay for the self-cleavage efficiency of the 2A peptides. *S. cerevisiae* Y2H-BD carried an empty vector pGBKT7 as a positive control. *S. cerevisiae* Y2H-BH carried NifB-NifH fusion without 2A as a native control. *S. cerevisiae* Y2H-BP2AH carried NifB-P2A-NifH fusion. *S. cerevisiae* Y2H-BT2AH carried NifB-T2A-NifH fusion. *S. cerevisiae* Y2H-BE2AH carried NifB-P2A-NifH fusion and *S. cerevisiae* Y2H-BF2AH carried NifB-F2A-NifH fusion. All yeast strains grown on SC-Trp and SC-Trp-His-Ade agar plates.


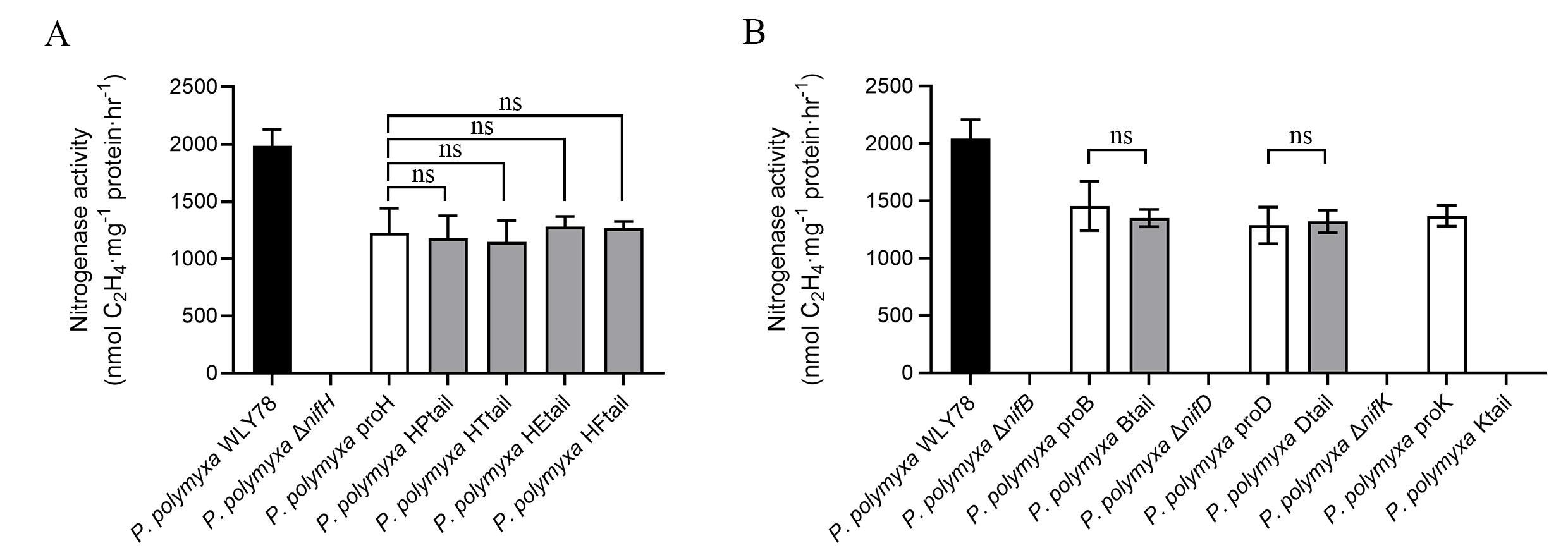


**Figure S2**. Assessment of tolerance of NifB, NifH, NifD and NifK to 2A tails. (A) Nitrogenase activities of wild-type *P. polymyxa* WLY78, Δ*nifH* mutant strain and complemented strains. *P. polymyxa* proH carried *nifH* without 2A tail. *P. polymyxa* Ptail carried *nifH* with P2A peptide. *P. polymyxa* Ttail carried *nifH* with T2A peptide, *P. polymyxa* Etail carried *nifH* with E2A peptide , and *P. polymyxa* Ftail carried *nifH* with F2A peptide. (B) Nitrogenase activities of *P. polymyxa* WLY78, mutants (Δ*nifB*, Δ*nifD* and Δ*nifK*) and complemented strains. *P. polymyxa* proB carried *nifB* without 2A peptide. *P. polymyxa* Btail carried *nifB* with P2A peptide. *P. polymyxa* proD prod carried *nifD* without 2A peptide. *P. polymyxa* Dtail carried *nifD* with P2A peptide. *P. polymyxa* proK carried *nifK* without 2A peptide. *P. polymyxa* Ktail carried *nifK* with P2A peptide. The *P* value was determined by the one-way ANOVA (n=3), ns: non-significant. Error bars indicated SEM.

**Table S1.** Stains used in this study

| Strains | Genotype or phenotype | Reference or source |
| --- | --- | --- |
| *E. coli* JM109 | *recA*1, *endA*1, *gyrA*96, *thi*-1, *hsdR*17, *supE*44, *relA*1, Δ(*lac-proAB*)/F’[*traD*36, *proAB*+, *lacIq*, *lacZΔM*15] | TaKaRa Co. China |
| *Saccharomyces cerevisiae* YSG50 | *MATa*, *ade*2-1, *ade*3-22, *ura*3-1, *his*3-11, 15, *trp1*-1, *leu*2-3,112 and *can*1-100 | (Shao et al., 2009) |
| *S. cerevisiae* Nif | A derivative of *S. cerevisiae* YSG50 carrying plasmids pRS423-*GAL1p* with (*nifB*-P2A-*nifH*-T2A-*nifD*-E2A-*nifK*) and pRS424-*GAL1p* with (*nifE*-F2A-*nifN*-T2A-*nifX*-F2A-*hesA*-P2A-*nifV*) control of *GAL1* promoter | This study |
| *S. cerevisiae* USHp | A derivative of *S. cerevisiae* YSG50 carrying plasmid pRS423-*GAL1p* with (*nifU*-P2A-*nifS*-P2A-*nifH*-His tag) under control of *GAL1* promoter | This study |
| *S. cerevisiae* USHt | A derivative of *S. cerevisiae* YSG50 carrying plasmid pRS423-*GAL1p* with (*nifU*-T2A-*nifS*-P2A-*nifH*-His tag) under control of *GAL1* promoter | This study |
| *S. cerevisiae* BH | A derivative of *S. cerevisiae* YSG50 carrying plasmid pRS423-BH with (*nifB-nifH*) under control of *GAL1* promoter | This study |
| *S. cerevisiae* P2A | A derivative of *S. cerevisiae* YSG50 carrying plasmid pRS423-BP2AH with (*nifB*-P2A-*nifH*) under control of *GAL1* promoter | This study |
| *S. cerevisiae* T2A | A derivative of *S. cerevisiae* YSG50 carrying plasmid pRS423-BT2AH with (*nifB*-T2A-*nifH*) under control of *GAL1* promoter | This study |
| *S. cerevisiae* E2A | A derivative of *S. cerevisiae* YSG50 carrying plasmid pRS423-BE2AH with (*nifB*-E2A-*nifH*) under control of *GAL1* promoter | This study |
| *S. cerevisiae* F2A | A derivative of *S. cerevisiae* YSG50 carrying plasmid pRS423-BF2AH with (*nifB*-F2A-*nifH*) under control of *GAL1* promoter | This study |
| *S. cerevisiae* Y2H Gold | *MATα*, *trp*1-901, *leu*2-3, 112, *ura*3-52, *his*3-200, *Aur*1-C and *mel*1 | TaKaRa Co. China |
| *S., cerevisiae* Y2H-BD | A derivative of *S. cerevisiae* Y2H Gold carrying an empty vector pGBKT7 with *Gal4* BD domain under control *ADH*1 promoter | This study |
| *S. cerevisiae* Y2H-BH | A derivative of *S. cerevisiae* Y2H Gold carrying recombinant plasmid pGBKT7-BH-AD with NifB-NifH fusion without 2A peptide | This study |
| *S. cerevisiae* Y2H-BP2AH | A derivative of *S. cerevisiae* Y2H Gold carrying recombinant plasmid pGBKT7-BP2AH-AD with NifB-P2A-NifH fusion | This study |
| *S. cerevisiae* Y2H-BT2AH | A derivative of *S. cerevisiae* Y2H Gold carrying recombinant plasmid pGBKT7-BT2AH-AD with NifB-T2A-NifH fusion | This study |
| *S. cerevisiae* Y2H-BE2AH | A derivative of *S. cerevisiae* Y2H Gold carrying recombinant plasmid pGBKT7-BE2AH-AD with NifB-E2A-NifH fusion | This study |
| *S. cerevisiae* Y2H-BF2AH | A derivative of *S. cerevisiae* Y2H Gold carrying recombinant plasmid pGBKT7-BF2AH-AD with NifB-F2A-NifH fusion | This study |
| *P. polymyxa* WLY78 | N_2_-fixing bacterium (wild-type) | (Wang et al., 2013) |
| *P. polymyxa* Δ*nifH* | A *nifH* deletion mutant strain of *P. polymyxa* WLY78 | This study |
| *P. polymyxa* proH | A derivative of *P. polymyxa* Δ*nifH* carrying plasmid pRN5101 with *nifH* under control of *nif* promoter (*Pnif*) | This study |
| *P. polymyxa* Ptail | A derivative of *P. polymyxa* Δ*nifH* carrying plasmid pRN5101 with *nifH*-Ptail under control of *nif* promoter (*Pnif*) | This study |
| *P. polymyxa* Ttail | A derivative of *P. polymyxa* Δ*nifH* carrying plasmid pRN5101 with *nifH*-Ttail under control of *nif* promoter (*Pnif*) | This study |
| *P. polymyxa* Etail | A derivative of *P. polymyxa* Δ*nifH* carrying plasmid pRN5101 with *nifH*-Etail under control of *nif* promoter (*Pnif*) | This study |
| *P. polymyxa* Ftail | A derivative of *P. polymyxa* Δ*nifH* carrying plasmid pRN5101 with *nifH*-Ftail under control of *nif* promoter (*Pnif*) | This study |
| *P. polymyxa* Δ*nifB* | A *nifB* deletion mutant strain of *P. polymyxa* WLY78 | This study |
| *P. polymyxa* proB | A derivative of *P. polymyxa* Δ*nifB* carrying plasmid pRN5101 with *nifB* under control of *nif* promoter (*Pnif*) | This study |
| *P. polymyxa* Btail | A derivative of *P. polymyxa* Δ*nifB* carrying plasmid pRN5101 with *nifB-*Ptail under control of *nif* promoter (*Pnif*) | This study |
| *P. polymyxa* Δ*nifD* | A *nifD* deletion mutant strain of *P. polymyxa* WLY78 | This study |
| *P. polymyxa* proD | A derivative of *P. polymyxa* Δ*nifD* carrying plasmid pRN5101 with *nifD* under control of *nif* promoter (*Pnif*) | This study |
| *P. polymyxa* Dtail | A derivative of *P. polymyxa* Δ*nifD* carrying plasmid pRN5101 with *nifD-*Ptail under control of *nif* promoter (*Pnif*) | This study |
| *P. polymyxa* Δ*nifK* | A *nifK* deletion mutant strain of *P. polymyxa* WLY78 | This study |
| *P. polymyxa* proK | A derivative of *P. polymyxa* Δ*nifK* carrying plasmid pRN5101 with *nifK* under control of *nif* promoter (*Pnif*) | This study |
| *P. polymyxa* Ktail | A derivative of *P. polymyxa* Δ*nifK* carrying plasmid pRN5101 with *nifK-*Ptail under control of *nif* promoter (*Pnif*) | This study |

**Table S2.** The codon-optimized sequences used in this study

| **1.** ***nifB-P2A-nifH-T2A-nifD-E2A-nifK*** |
| --- |
| *nifB*  ATGGACTCTTTGGCTGATTTGTCTGAAACCCCATTGGCATTGGAAACTTTGAGACGTCATCCATGTTATAACGAAGAGGCACATAGATATTTTGCTAGAATCCATTTGCCAGTTGCCCCAGCATGCAATATTCAGTGCCATTATTGCAACAGAAAATTCGATTGCGTCAATGAATCTAGACCAGGTGTTGTTAGTGAATTGTTGACTCCAGAGCAGGCTGCTTCTAAGACCTATGGTGTTGCTGCACAGTTGATGCAGTTGTCCGTTGTCGGTATTGCTGGACCTGGAGATCCATTGGCCAATGCTGAGGCAACCTTCGATACCTTCAGAAGAGTCAGAGAGACAGTTAAGGACGTCATTTTCTGTTTGTCTACTAATGGTTTGACTTTGATCAGACATATCGACAGAATTGTTGAGTTGGGTATTTCTCATGTCACTATCACTATCAATGCTGTTGATCCAGTTGTTGGTTCTAGAATTTATGGATGGGTCTACGATGAAGGAAAAAGATATGCTGGTGAGGAGGCCGCAAGATTGTTGATTGACAGACAGTTGGCAGGTTTGAAGATGTTGGCTTCTAGAGGTGTTTTGTGCAAGGTTAACTCTGTTTTGATTCCAGAAGTCAATGATGCCCATTTGCCAGAGGTTGCTAGAGTTGTCAAGGAGCACGGTGCTGTTTTGCACAACATTATGCCATTGATCATCGCACCTGGTTCTAGATATGAGCAGGAAGGTATGAGAGCACCAAGACCAAGATTGGTCAGACAGTTGCAGGAGCAATGTGCTGAAGCTGGAGCTGTCATTATGAGACATTGCAGACAGTGCAGAGCTGATGCTATTGGATTGTTGGGTGAGGATAGAAATCAGGATTTTACATGGGAGAACATTGCTGCTGCTCCTCCAATGGATGAAGAGGCAAGAGCACAATTTCAGAAAGAATTGGATGAGAAGGTTAGAGTTAGAATGGAAAGAAAGGAGGGACAATCTCACCACAAACAACCATCAACCGGTGCTGGTTGTTCTTGCCCATTGTCTGGTGATAAGCCTGAGGCTTCTTTTACCTCAAAGCCAGTCTTGATCGCTGTTGCTAGTAGAGGTGGAGGTAAGGTTAATCAGCATTTCGGTAGAGCCAAGGAATTTATGATCTATGAATCTGACGGAACCATCGTTAATTTCATTGGTATTAGAAAGGTTCAATCCTACTGTCACGGTAAAGCCGATTGCAATGGAGATAAGGCCGAGACTATCAAGGAGATCTTGTCCATGGTTCATGATTGTGCATTGTTGTTGTCCTCCGGTATTGGTGAAGCCCCAAAAGAGGCATTGCAGGAAGCTGGTGTTTTGCCTATTGTTTGCGGTGGTGATATTGAGGAGTCCGTTTTGGAATATGTTAAATTTTTGAGATATATGTATCCTGTTCAGACTGGTAAGGGAAGTAAGAGAAATAAGGGAGTTAAGGGTAATCATTCTGATTTGCCAATTGAACATTTTGGAGGT  -gctactaacttctctttgcttaagcaagccggagacgttgaggaaaacccaggtcct (P2A)- |
| *nifH*  ATGAGACAAATTGCTTTTTACGGTAAGGGTGGTATCGGTAAATCTACAACCTCTCAGAATACATTGGCTCAATTGGCTACCAAATTCAAACAAAAAATTATGATCGTTGGTTGTGATCCAAAGGCAGACTCCACCAGATTGATTTTGAATACTAAGGCCCAACAGACCGTTTTGCATTTGGCAGCTGAAAGAGGTACTGTTGAGGACTTGGAATTGGAGGATGTTGTCCAGAAGGGTTTCGGTGATATTTTGAACGTTGAATGCGGTGGTCCAGAGCCAGGTGTCGGTTGTGCAGGAAGAGGTATCATCACAGCCATTAATTTTTTGGAGGAAGAGGGTGCCTACGAAGGTTTGGATTTCGTTTCCTACGATGTTTTGGGTGACGTCGTTTGCGGTGGTTTCGCCATGCCAATCAGAGAGAAGAAGGCTCAGGAAATCTACATCGTTTGCTCAGGTGAGATGATGGCTATGTACGCTGCCAACAATATTGCTAGAGGTATCTTGAAGTATGCCAACTCTGGTGGTGTTAGATTGGGTGGTTTGATCTGCAACTCTAGAAATACTGACTTGGAAGCTGAATTGATCACAGAGTTGGCAAGAAGATTGAACACTCAGATGATCCACTTTTTGCCAAGAGACAATGTTGTTCAGCACGCTGAGTTGAGAAGAATGACCGTTACCCAATATAACCCAGAACATAAGCAGGCTGCTGAGTATGAAGAGTTGGCAGGTAAGATTTTGAATAATGACATGTTGACTGTTCCAACTCCAATTTCCATGGAAGATTTGGAGGATTTGTTGATGGAGTTCGGTATTATTGAGGATGAAGAAACCGCAATTAACAAAGCTGAGGCTTCCGGTCAG  - gaaggtagaggttctttgttgacttgtggtgatgttgaagaaaacccaggtcca (T2A)- |
| *nifD*  ATGTCTAGTATTGTTGATAAGGGTAAGCAGATCGTTGAGGAGATTTTGGAGGTTTATCCAAAGAAGGCCAAGAAGGATAGAACCAAGCATTTTGAGATCGCTGATGAGGAGCTTGTTAACTGCGGAACCTGTTCCATCAAGTCCAACATGAAATCAAGACCTGGTGTCATGACAGCAAGAGGTTGTGCTTATGCAGGTTCCAAGGGTGTTGTTTGGGGTCCAATTAAAGACATGGTTCACATTTCTCATGGTCCAATCGGTTGCGGACAGTACAGTTGGGGAACCAGAAGAAATTATGCTAATGGTATTTTGGGAATCGATAATTTTACCGCCATGCAGATTACATCTAATTTTCAGGAAAAAGATATCGTTTTCGGTGGAGATAAGAAGTTGGAGGTTATCTGCAGAGAAATTAAGGAGATGTTCCCATTGGCTAAGGGTATCTCCGTTCAATCTGAATGTCCAGTCGGATTGATTGGTGATGATATCGGTGCCGTTGCCAAGAAGATGACAGAGGAGTTGGGTATTCCAGTCATTCCTGTTAGATGTGAGGGTTTTAGAGGTGTTAGTCAGTCTTTGGGTCATCACATTGCCAATGATGCTATCAGAGATTTTTTGATGGGTAGAAGAGAATTGAAGGAGTGCGGTCCTTATGATGTCTCCATTATCGGAGACTACAATATCGGTGGTGATGCCTGGGCTTCTAGAATTTTGTTGGAGGAAATGGGATTGAGAGTCATTGCTCAGTGGTCTGGTGACGGTACTATCAATGAGTTGGGTATTGCTCATAAATCCAAGTTGAACTTGATCCATTGTCATAGATCCATGAATTATATGTGCACAACAATGGAGCAGGAATACGGAATCCCATGGATGGAATATAACTTCTTCGGTCCAACCAAGACTATGGAGTCTTTGAGAGCTATTGCTGCCAGATTCGACGAGACTATTCAGGAAAAATGTGAGCAGGTCATCGCCCAATATATGCCACAGATGGAGGCTGTCATCAGAAAATATAGACCAAGATTGGAAGGTAAAAAGGTTATGCTTTTGATTGGTGGTTTGAGAGCAAGACATACCATCGGTGCCTATGAGGATTTGGGTATGGAAATTGTTGCTACAGGTTATGAATTTGCCCATAAGGATGATTACGAAAAGACTTTTCCAGATGTTAAAGAAGGAACCATTTTGTACGATGATCCAACTGCATATGAGTTGGAGGAATTGGCCCAGAGATTGAATATTGACTTGATGGGTGCCGGAGTCAAGGAGAAATACGTTTATCACAAAATGGGTATTCCATTCAGACAAATGCACTCCTGGGATTACTCTGGTCCTTATCATGGTTTTGACGGTTTTAAGATTTTTGCAAGAGATATGGATATGACCATTAACAGTCCAGTTTGGTCTTTGTTGCCATCAAGACAGACTGCTGAGGTTCCAGTT  - caatgtactaactacgctcttttgaagttggccggagatgtcgagtctaaccctggacca (E2A)- |
| *nifK*  ATGGAGCCTGCTGCCTTGACTGCAGACTGCGGAGGTGCCGGTATGTCTGAAAGACCAAATATTGTCGATCACAATCAGTTGTTTAGACAGGATAAATATGTTAGACAGAGAGAAGAAAAAAGAGCCTTCGAGGCCCCATGTTCTCCAGAGGAGGTTACCGACACCTTGGAGTACACCAAGACCAAGGAATACAAAGACAAGAATTTTGCCAGAACAGCCGTTGTCGTTAATCCAGCCAAGGCTTGTCAGCCATTGGGAGCTGTTATGGCTGCATTGGGTTTCGAAAAAACTTTGCCATTCATTCATGGTTCACAGGGTTGTACTGCTTATTTTAGAAGTCATTTGGCCAGACACTTCAAAGAGCCTGTTCCTGCCGTCTCCACCTCTATGACCGAGGATGCCGCCGTTTTCGGTGGTATGAGAAACTTGATTGACGGTATTGAGAACTGCATTGCCTTGTATCAGCCAGAGATGATTGCTGTTTGCACTACCTGTATGGCAGAGGTTATCGGTGATGATTTGTCTGCCTTCTTGGCCAATGCCAGACAGGAGGGAGTCTTGCCTGAGGATATGCCAGTTCCTTTTGCCAATACCCCATCTTTCTCTGGTTCACATATTACAGGTTATGACGCCATGTTGAGATCTGTTTTGGAGACTTTGTATAACAAGTCAGGTAGAACTGCTCAGCCTGGTCATGAATTGAAGTTGAATGTTTTGTTGGGTTTTGACGGTTATACTGGTAATTTTGCTGAAATGAGAAGAATGTTGGGTATGTTCGGTGCTACTTATACCATTTTGGGTGACCACTCTAGTAATTTTGATTCAGGTGCCACTGGAGAGTACTCTTACTATTACGGTGGAACTCCATTGGAGGATGTTCCTAAGGCCGCAGATGCTGCCGGTACTTTGGCTATTCAGCAGTACTCTTTGAGAAAAACATTGGGTTATATGAAGCAAACCTGGGGTCAGCAGGTTTCCTCCATCTCCACACCATTGGGTATCAGAGCTACAGATAGATTGTTGGAGGAGATTTCTAGATTGTCTGGAAGAGAAATTCCAGAGGCATTGAAGCAGGAGAGAGCCAGAATTGTTGATGCCATGATGGATTCACATGCTTATTTGCACGGTAAAAGAGTTGCTATGGCAGGTGACCCAGACATGTTGATCGGTTTGATTGGTTTTTGTTTGGAGTTGGGTATGGAGCCAGTTCATATTGTTTGCTCCAATGGTGACAGAAAATTTGAGAAGGAAGCAGAGTTGTTGTTGAAGTCCTCTCCTTACGGTGCAGAAGCCACTGTTCATTCCGGTCAGGATTTGTGGCATATGAGATCTTTGTTGTTCCAGGACCCAGTTGACTTGGCTATTGGTTCTTCCCATTTGAAGTTTGCAGCTAAAGAGGCTGAAATTCCTTTGTTGAGAGTTGGTTTTCCAATCTTCGACAGACATCATTTGCATAGATATCCAATTATCGGTTACCAGGGTGCTTTGAATTTGTTGACCCAATTCGTTAATACCATTTTGGATGTCATGGAGGAGCAGGCTCCAGATCATTCTTTTGATTTGGTTAGATAA |
| **2. *nifE-P2A-nifN-T2A-nifX-E2A-hesA-F2A-nifV*** |
| *nifE*  ATGGAGCCAGCTGTTTCTAACGGAAGATTGGAGGTTTCCTGCGGTAATAAAATTCCAAAATCTACTCCATGTCCAAGACCTGTTCCAGGAGAGGCTTCTGGTGGTTGCTCCTTTGACGGTGCCCAGATTACATTGATCCCAATTGCAGATGCTGCTCATTTGGTTCACGGTCCAATTGCTTGTTTGGGTAATTCTTGGGAGTCTAGAGGTAGTTTGTCCTCTGGTCCAGAGTTGTCTGCTTATGGTTTCACTACTGATTTGGGAGAACAGGACATCATTTTTGGTAGTGAACAGAAGTTGCATGAATCTATCAGATACATTGTCTCTAGATTTGCTCCTCCAGCTGTTTTTGTCTATACCACATGTGTCACAGCCTTGACTGGTGAAGATATCGAGGGTGTTTGCAAGGCTGAATCTGAGAGATTGGGTACTCCAATCATTCCAGTTAACAGTCCAGGATTTGTTGGTAGTAAGAATTTGGGAACCAGATTGGCCGGAGATGTTTTGTTCCAGCATATTATCGGTTCTACCGAGCCAGAACAGACAACCTCCCATGATATCAATTTGATTGGTGAATACAATATTGCTGGTGAGATGTGGCATATCGAGAGATTGATGCAGCAGGCTGGAATGAGTATCTTGTCCAGAATTACCGGTGACGGTAGATTCAGAGAGGTTGGTTGGGCTCACAGAGCCAAGGCCAACATGGTCGTTTGCTCTAGAGCTTTGTTGGGTTTGGCAGTCCAAATGGAGAGAAAATACGGTATTCCTTATTTTGAAGGTTCATTTTATGGAGCAAAGGAGACTAGTTATTCCTTGAGACAGATGGCTTACTTGACCGGAGATAGAGATGTTGAGAGAAGAGTTGATAAGTTGGCCGCAAGAGAGGAAATGAGATTGTCTTTGGAGTTGGAGCCATACAGAAAGCAGTTGAAAGGAAAGAGAGCAGTTTTGTATACCGGTGGTGTTAAGTCTTGGTCTGTCATTACTGCTTTGCAGGAGTTGGGTATTAAGGTTGTTGGTGTTGGTACTAACAAGTCTACTGCCGAGGATGTTTCCAGAATTGCTGACAGAATCGGTGATGATGCAGAATACATCCCAGAAGGAGGTGCCAGACAGATTTTGAAAACCGTTAGATCTAGAAAGGCAGACATGGTCATTGCCGGAGGTAGAAACATGTATATGGCTTTGAAGGAACAGATTCCTTTTGTTGACATCAATCAAGAGAGACACAAAGCCTATGCTGGTTATGACGGTTTGTTGTCTTTGGCTAAACAGTTGGTTCATACTTTGCAGCATCCAGTTTGGGAGTTGACCGCCAAATTGGCTCCATGGGAGGAGGAGACTGAATTTGCTGAT  -gctactaacttctctttgcttaagcaagccggagacgttgaggaaaacccaggtcct (P2A)- |
| *nifN*  ATGTATAGATCAATGCCTTTGTTGCACGGTGCTCAGGGTTGCTCTGCCTTCTCCAAGGCTTTGTTGACTAGACATTTTAGAGAGCCAATTGCCGTTCAGACCTCTGCTTTGCAAGAGATGGACGTTATTTTTGATGCAGACAGAAATTTGGAGGAGGCTTTGGATCATATCTGGTCCAAACACCATCCAGATGTCATCGGTGTTATCTCTACTGCCTTGACTGAGGTTGCAGGTGTTGACTTTCAGTCTAGAGTTAAGGCTTTCAAGAGAGAAAGAGCATTGAAGGACAGTTTGTTGTTTTCTGTTTCTTTGCCTGATTTTCACGGTTCTTTGGAGACTGGTTACTCTAGTACAGTTGAGTCATTGATGGATGCCGTTTTGGGTTTGGCCGGTGGTAAGTCCCCAAAAAAACAGAGAAGAACTCAGGTCAATTTGTTGCCAGCTTCTTATTTGACTGCCGGAGATGTCATGGAAATCAAGGATATTATCGCTTCCTTCGGTTTGGAGGTTATTACTTTGCCAGATATTTCCACTTCCTTGTCCGGTCACTTGTTGACAGGTTTTTCCCCTTTGACTAGAGGTGGTACTCCATTGGATTCAGCCTGCCAGATGTTGGAGTCCTCCTGCACCATTGCCATTGGTGCTTCTATGGAAAGACCAGCTAGAAGATTGACTCATGCTGCAGGTATTCCATACCACTTGTTCGCTGGTTTGTCTGGTTTGGCCGCTAGTGATGCTTTCATTCATTTTTTGCAGAAAATCTCTAGAGAGCCAGCCCCAGTTAGATTCAGATGGCAGAGAGAAAATTTGTTGGACTCTATGTTGGATGCCCATTTCTATTATTCTGGTGCTTCTGCTGTTGTTGCTTTGGAACCAGATCATATGTTGTCTACCGCAGCCTGGTTGGAGGAGATGGGAGTTGAATTGAAGAGATTGATTACACCATGCTCTACTCCAGCATTGCAAAAGACAGAAAGAGAAGTCTGGATCGGTGACTTGGATGATGCAGAGGAGTCTGCTCAGGGTGTTGATTTGTGGATCTCTAACTCACATGGAAGAAAGGGAGCTGCAAGAGCTGGTGCCTCATTCGTTCCAGCAGGTTTGCCAGTTTATGACGAGTTGGGTGCCCACACATCCGTTTCTGTCGGATACAGAGGAACCATGGAGTGGGTTAACAAAGTTGGTAATGTTTTGTTGGCCGAGAGAGGTAGAGGAGGA  - gaaggtagaggttctttgttgacttgtggtgatgttgaagaaaacccaggtcca (T2A)- |
| *nifX*  ATGAAGGTTGCTTTTGCTACTGAGGACGGTGTTTTGGTTAATGCTCATTTTGGTCAGTCTCCAATGTTCACTATTTTCGAAATCAGACACTCAGGTGTTCAGTTCTTGGAGCATAGAAGAATTGCCTTGGGTTCTGATGAGAATGAGGCTGGTAAGATCGCCTCTAGAATTGGTTTGATCGAGGATTGTGCCTTGATCTTCTTGGTTCAGATTGGTGCTTCCGCCGCCGCTCAGGTTACCAAGAGAACCATTATGCCTGTTAAGGTTGCCTTCGGTTCTACCATTGAGGAGCAGGTTCAGAGATTGCAGAATATGTTGACTAGAAATCCACCAATGTGGTTGGCCAAAATCTTGCATGCTGAGGAGGGTTCTGGTAAAGCCGAATCA  - caatgtactaactacgctcttttgaagttggccggagatgtcgagtctaaccctggacca (E2A)- |
| *hesA*  ATGTTGAGAAGAGCAGCTGCAAAGCCAAATCATGAGCCATCCTGTAAGGAAGAGCAACCATATAGAGTTTTGAGATCCTGCAGACCAAATATCTTGAAGGCTGGAGCCGCACATGGAGGTGGTGGAAGAATGGTTCAATTGTTGGAGGACAGTAGATACGGTAGACAGTTGAAGTTGTTGGGAGTTGAAGGTCAGAACAGATTGAAGCAGGCTACTGTTATGGTTGCAGGTATCGGAGGATTGGGAGGTGCAGCTGCCATGTACTTGGCCGCTGCCGGAGTTGGAAAGTTGATTTTGGCCCATGAGGGTGTTATCCATTTGCCAGATATGAACAGACAGGTTTTGATGGACTCTGGAAGAATCGGTGAGGAAAGAATGGAGACTGCATTGCAGCATTTGCATAGAATCAATCCAGAAACCGAGTTGGAGGGTCACGCCCACAGAATCACTGAAGAATCCTCTGGACCATGGGTTGAGGCTTCTGATATCGTTATTGATGCAAGATATGACTTTCCAGAAAGATATGCTTTGAACAGATTGTGTGTTAGACATGGTAGACCAATGATTGAAGCTGCCATGTACGCCTATGAAGTTTCATTGATGACCATTGATCCAGGTAAGACTGCATGCTTGGAATGTTTGTACCCAGAAGGTGGACAGCCTTGGGAACCTTTGGGATTCCCAGTCTTGGGAGCCACCTCCGGTTTGATTGGTTGCATGGCTGCATTGGAAGCTGTCAAATGGATTACAGATGCTGGTAATTTGTTCACTGACAGAATGTACAGAATGAATGTTTTGGATATGTCCTCTTGCACCATTGCTGTCAAAAGAAACCCAAGATGTCCATGCTGCGGAACTGGAGGTGATACAGATGAGTCTGTTGCATATTTG  -gttaagcaaactttgaacttcgacttgcttaaattggctggtgacgttgaatctaacccaggacct (F2A)- |
| *nifV*  ATGAGTAGATTGCATATTTGTGATACTACACTTAGAGACGGAGAACAGGCTCCAGGTGTTGCCTTTTCAGCCGAGGAAAAAACTGAAATTGCCATCATGTTGGACTCTGCTGGTGTTGAGCAGGCTGAGATCGGAATCCCAGCAATGGGAAAGACTGAGTGCAGATCTATTGCCAGAATTGCTGCTTTGGGACTTCAGATGAAGTTGATGACCTGGAATAGAGCTGTTTTCACTGATATTGATGCAACTGAATCTACAGGTGTCGGTTGGGCCCATATTTCTGTTCCAGTTTCTACTGTTCAGATGAAGTCCAAGTTGGGTATGAATCCTGAGCAGGTTACTGAGTTGATCAGAAAGTCTGTCGATTACGCTTTGTGTAAAGGATTGACTGTTTCCGTTGGTTTTGAGGATGCTTCAAGAGCAGATGACTTGTTCCTTGAGCAGTTGGCTAATCAGTTGTATAGAGATGGTATCAGAAGATTCAGATATGCCGATACTTTGTCCGTTCACCATCCAGCTGCCATTGCTGCCAGAATTGACAGACTTGTTTCTAGAGTTCCACAGGATGTTGAGCTTGAGATTCACTGTCATAATGATTATGGTTTGGCTCTTGCCAATACCTTGGCAGCTTTGCAAGCTGGAGCTGTCTGGGCCAGTACCACTGTTTCTGGACTTGGTGAAAGAGCAGGTAATACCGCTTTGGAGGAGGTTGTTATGTCTTGGAGAGACTTGTATCAAGGAACCTGCTCTGTCAGACCAGAATTGTTGAACCCATTGGCTGCATTGGTTTCCAAAGCCTCCAACAGAATCATTCCTGAAGGTAAGCCAATTGTTGGAGACATGGTTTTCGCCCATGAATCCGGTATTCATATCAACGGTTTGTTGAAGGAGAGAGCCGCCTATCAGGCTCTTGATCCAACTGAGTTGGGTACTGACCATTCCTTCGTTTTGGGTAAGCATTCTGGTAGAAGTGCAGTTCAATATATGTTGGAGCAGGAAGGAATCGAGGCAGGTTCCGGTGAAATCAAGTTCTTGTTGGAGAGACTTAGATTGGTCGGTGAAGATCCAAAGAGAGTCATCCATTCTGCTGATTTGAGAAGATGGTTGCAGTATTATCCAGCAGAGTTGCCAAAATAA |

**Table S3.** Primers used in this study

| Genetic element 1 | Genetic element 2 | Primer sequence (5'→3') | Target |
| --- | --- | --- | --- |
| *nifB-nifH* | *nifB* | f1: GTGGATCCCCCGGGCTGCAGGAATTCATGGACTCT TTGGCTGATTTG | For construction of plasmid pRS423-BH |
|  |  | r1: GTAAAAAGCAATTTGTCTCATACCTCCAAAATGTTC AATTG |  |
|  | *nifH* | f2: CAATTGAACATTTTGGAGGTATGAGACAAATTGCTT TTTAC |  |
|  |  | r2: CTAATTACATGACTCGAGGTCGACCTACTGACCGGA AGCCTCAG |  |
| *nifB-P2A-nifH* | *nifB-P2A-nifH* | f1: GTGGATCCCCCGGGCTGCAGGAATTCATGGACTCTT TGGCTGATTTG | For construction of plasmid pRS423-BP2AH |
|  |  | r2: CTAATTACATGACTCGAGGTCGACCTACTGACCGGA AGCCTCAG |  |
| *nifB-T2A-nifH* | *nifB-T2A* | f1: GTGGATCCCCCGGGCTGCAGGAATTCATGGACTCTT TGGCTGATTTG | For construction of plasmid pRS423-BT2AH |
|  |  | r4: TCTTCGACATCACCACAAGTCAACAAAGAACCTCTA CCTTCACCTCCAAAATGTTCAAT |  |
|  | *T2A-nifH* | f5: TTTGTTGACTTGTGGTGATGTCGAAGAGAACCCTGGT CCAATGAGACAAATTGCTTTTT |  |
|  |  | r2: CTAATTACATGACTCGAGGTCGACCTACTGACCGGA AGCCTCAG |  |
| *nifB-E2A-nifH* | *nifB-E2A* | f1: GTGGATCCCCCGGGCTGCAGGAATTCATGGACTCT TTGGCTGATTTG | For construction of plasmid pRS423-BE2AH |
|  |  | r6: ACATCTCCGGCCAACTTCAAAAGAGCGTAGTTAGTA CATTGACCTCCAAAATGTTCAAT |  |
|  | *E2A-nifH* | f7: TTTGAAGTTGGCCGGAGATGTCGAGTCTAACCCTGG ACCAATGAGACAAATTGCTTTTT |  |
|  |  | r2: CTAATTACATGACTCGAGGTCGACCTACTGACCGGA AGCCTCAG |  |
| *nifB-F2A-nifH* | *nifB-F2A* | f1: GTGGATCCCCCGGGCTGCAGGAATTCATGGACTCTT TGGCTGATTTG | For construction of plasmid pRS423-BF2AH |
|  |  | r8: ACCAGCCAACTTCAACAAATCAAAGTTCAAAGTTT GCTTAACACCTCCAAAATGTTCAA |  |
|  | *F2A-nifH* | f9: GTTGAAGTTGGCTGGTGATGTTGAATCTAACCCAGG TCCAATGAGACAAATTGCTTTTT |  |
|  |  | r2: CTAATTACATGACTCGAGGTCGACCTACTGACCGGA AGCCTCAG |  |
| *AD (GAL4 activation domain)* | *AD* | f10: GCCATGGAGGCCGAATTCGCCAATTTTAATCAAAG TG | For construction of plasmid pGBKT7-AD |
|  |  | r10: CGCTGCAGGTCGACGGATCCCTAGATCTCTTTTTT TGGGTTTG |  |
| *nifB-nifH* | *nifB-nifH* | f11: GCCATGGAGGCCGAATTCATGGACTCTTTGGCTGAT | For construction of plasmid pGBKT7-BHAD |
|  |  | r11: CACTTTGATTAAAATTGGCCTGACCGGAAGCCTCAG C |  |
| *nifB-P2A-nifH* | *nifB-P2A-nifH* | f11: GCCATGGAGGCCGAATTCATGGACTCTTTGGCTGAT | For construction of pGBKT7-BP2AHAD |
|  |  | r11: CACTTTGATTAAAATTGGCCTGACCGGAAGCCTCA GC |  |
| *nifB-T2A-nifH* | *nifB-T2A-nifH* | f11: GCCATGGAGGCCGAATTCATGGACTCTTTGGCTGAT | For construction of plasmid pGBKT7-BT2AHAD |
|  |  | r11: CACTTTGATTAAAATTGGCCTGACCGGAAGCCTCA GC |  |
| *nifB-E2A-nifH* | *nifB-E2A-nifH* | f11: GCCATGGAGGCCGAATTCATGGACTCTTTGGCTGAT | For construction of plasmid pGBKT7-BE2AHAD |
|  |  | r11: CACTTTGATTAAAATTGGCCTGACCGGAAGCCTCA GC |  |
| *nifB-F2A-nifH* | *nifB-F2A-nifH* | f11: GCCATGGAGGCCGAATTCATGGACTCTTTGGCTGAT | For construction of plasmid pGBKT7-BF2AHAD |
|  |  | r11: CACTTTGATTAAAATTGGCCTGACCGGAAGCCTCA GC |  |
| *nifH_up stream_-nifH_down stream_* | *nifH_up stream_* | f12: CGGCCACGATGCGTCCGGCGTAGAGCGCACGACT GTTGATTGAC | For construction of plasmid pRN5101-ΔH |
|  |  | r12: TGCTCATTTGGCCTCAGCCTCCAAAATGTTC |  |
|  | *nifH_down_* | f13: TTTTGGAGGCTGAGGCCAAATGAGCAGTATTGTGG |  |
|  |  | r13: GACTGCGCAAAAGACATAATCGATAGCAGCCCGCC AATCAGAAG |  |
| *Pnif-nifH* | *Pnif* | f14: CACGATGCGTCCGGCGTAGAGGATCCGCGGAGAC TATTTCCCA | For construction of pRN5101-proH |
|  |  | r14: GTAAAAAGCAATTTGTCTCATTCATTCCCTCCTCT CTACGT |  |
|  | *nifH* | f15: ACGTAGAGAGGAGGGAATGAATGAGACAAATTGC TTTTTAC |  |
|  |  | r15: TGCGCAAAAGACATAATCGATAAGCTTTCACTGAC CGGAAGCCTCAGC |  |
| *Ptail-pRN5101-Pnif-nifH-Ptail* | *Ptail-pRN5101-Pnif-nifH-Ptail* | f16: CTTAAGCAAGCCGGAGACGTTGAGGAAAACCCAGG TCCTTAAAAGCTTATCGATTATGT | For construction of plasmid pRN5101-Ptail |
|  |  | r16: TCAACGTCTCCGGCTTGCTTAAGCAAAGAGAAGTT AGTAGCCTGACCGGAAGCCTCAGC |  |
| *Ttail-pRN5101-Pnif-nifH-Ttail* | *Ttail-pRN5101-Pnif-nifH-Ttail* | f17: TTTGTTGACTTGTGGTGATGTCGAAGAGAACCCTGG TCCATAAAAGCTTATCGATTATG | For construction of pRN5101-Ttail |
|  |  | r17: TCTTCGACATCACCACAAGTCAACAAAGAACCTCT ACCTTCCTGACCGGAAGCCTCAGC |  |
| *Etail-pRN5101-Pnif-nifH-Etail* | *Etail-pRN5101-Pnif-nifH-Etail* | f18: TTTGAAGTTGGCCGGAGATGTCGAGTCTAACCCTG GACCATAAAAGCTTATCGATTATG | For construction of pRN5101-Etail |
|  |  | r18: ACATCTCCGGCCAACTTCAAAAGAGCGTAGTTAGT ACATTGCTGACCGGAAGCCTCAGC |  |
| *Ftail-pRN5101-Pnif-nifH-Ftail* | *Ftail-pRN5101-Pnif-nifH-Ftail* | f19: GTTGAAGTTGGCTGGTGATGTTGAATCTAACCCAG GTCCATAAAAGCTTATCGATTATG | For construction of plasmid pRN5101-Ftail |
|  |  | r19: CCAGCCAACTTCAACAAATCAAAGTTCAAAGTTTG CTTAACCTGACCGGAAGCCTCAGC |  |
| *nifB_up stream_-nifB_down stream_* | *nifB_up stream_* | f20: ACGATGCGTCCGGCGTAGAGGATCCATGAAGGTGG CACTTACG | For construction of plasmid pRN5101-ΔB |
|  |  | r20: GCACAGGATAATAGCGATGTGCCTCTTC |  |
|  | *nifB_down_* | f21: ACATCGCTATTATCCTGTGCAGACGGGTAAG |  |
|  |  | r21: CGCAAAAGACATAATCGATAAGCTTTGCTTGGTC CGATCCTTC |  |
| *Pnif-nifB* | *Pnif-nifB* | f14: CACGATGCGTCCGGCGTAGAGGATCCGCGGAGAC TATTTCCCA | For construction of plasmid pRN5101-proB |
|  |  | r22: TGCGCAAAAGACATAATCGATAAGCTTTCAGCCTC CAAAATGTTC |  |
| *Ptail-pRN5101-Pnif-nifB-Ptail* | *Ptail-pRN5101-Pnif-nifB-Ptail* | f23: TTAAGCAAGCCGGAGACGTTGAGGAAAACCCAGGT CCTTAAAAGCTTATCGATTATGTC | For construction of plasmid pRN5101-Btail |
|  |  | r23: TCAACGTCTCCGGCTTGCTTAAGCAAAGAGAAGTT AGTAGCGCCTCCAAAATGTTCAAT |  |
| *nifD_up stream_-nifD_down stream_* | *nifD_up stream_* | f24: GATGCGTCCGGCGTAGAGGATCCTAAGGGAAGTA AGCGTAATA | For construction of pRN5101-ΔD |
|  |  | r24: TGCATTTGACGGCTCATTTGGCCTATTCC |  |
|  | *nifD_down_* | f25: CCAAATGAGCCGTCAAATGCACTCCTGG |  |
|  |  | r25: CAAAAGACATAATCGATAAGCTTATGGAGGACAC CTGCTGC |  |
| *Pnif-nifD* | *Pnif* | f14: CACGATGCGTCCGGCGTAGAGGATCCGCGGAGAC TATTTCCCA | For construction of plasmid pRN5101-proD |
|  |  | r26: ATCCACAATACTGCTCATTCATTCCCTCCTCTCTAC GT |  |
|  | *nifD* | f27: ACGTAGAGAGGAGGGAATGAATGAGCAGTATTGTG GAT |  |
|  |  | r27: TGCGCAAAAGACATAATCGATAAGCTTTCATACCG GCACCTCCGC |  |
| *Ptail-pRN5101-Pnif-nifD-Ptail* | *Ptail-pRN5101-Pnif-nifD-Ptail* | f28: TTAAGCAAGCCGGAGACGTTGAGGAAAACCCAGG TCCTTAAAAGCTTATCGATTATGTC | For construction of plasmid pRN5101-Dtail |
|  |  | r28: TCAACGTCTCCGGCTTGCTTAAGCAAAGAGAAGT TAGTAGCTACCGGCACCTCCGCAG |  |
| *nifKup stream-nifKdown stream* | *nifKup stream* | f29: CGATGCGTCCGGCGTAGAGGATCCTTCCGGTCATT CCTGTAC | For construction of plasmid pRN5101-ΔK |
|  |  | r29: CACCCTGGTAGCCGATAATACATGGGGCCTCGAAG GC |  |
|  | *nifKdown* | f30: GCCTTCGAGGCCCCATGTATTATCGGCTACCAGGG TG |  |
|  |  | r30: CAAAAGACATAATCGATAAGCTTAAGCCATCTGCC GCAAGG |  |
| *Pnif-nifK* | *Pnif* | f14: CACGATGCGTCCGGCGTAGAGGATCCGCGGAGACT ATTTCCCA | For construction of plasmid pRN5101-proK |
|  |  | r31: GAGGGCAGCAGGCTCCATTCATTCCCTCCTCTCTA CGT |  |
|  | *nifK* | f32: ACGTAGAGAGGAGGGAATGAATGGAGCCTGCTGCC CTC |  |
|  |  | r32: TGCGCAAAAGACATAATCGATAAGCTTTTAGCGCA CCAGATCAAAGCT |  |
| *Ptail-pRN5101-Pnif-nifK-Ptail* | *Ptail-pRN5101-Pnif-nifK-Ptail* | f33: TTAAGCAAGCCGGAGACGTTGAGGAAAACCCAGGT CCTTAAAAGCTTATCGATTATGTC | For construction of plasmid pRN5101-Ktail |
|  |  | r33: TCAACGTCTCCGGCTTGCTTAAGCAAAGAGAAGTT AGTAGCGCGCACCAGATCAAAGCT |  |
| *nifB-P2A-nifH-T2A-nifD-E2A-nifK* | *nifB-P2A-nifH-T2A-nifD-E2A-nifK* | f34: ATCGAATTCATGGACTCTTTGGCTGATTTG | For construction of plasmid pRS423-c1 |
|  |  | r34: CGCGTCGACTTATCTAACCAAATCAAAAG |  |
| *nifE-F2A-nifN-T2A-nifX-F2A-hesA-P2A-nifV* | *nifE-F2A-nifN-T2A-nifX-F2A-hesA-P2A-nifV* | f35: CGCGAATTCATGGAGCCAGCTGTTTCTAAC | For construction of plasmid pRS424-c2 |
|  |  | r35: ATCGTCGACTTATTTTGGCAACTCTGCTG |  |
| *nifU-P2A-nifS-P2A-nifH* | *nifU-P2A* | f36: GTGGATCCCCCGGGCTGCAGGAATTCATGTGGAA CTACTCAGAAAAAG | For construction of plasmid pRS423-USHp |
|  |  | r36: TCAACGTCTCCGGCTTGCTTAAGCAAAGAGAAGTT AGTAGCGTTCACAGCTGCAGGAGC |  |
|  | *P2A-nifS-P2A* | f37: TGCTTAAGCAAGCCGGAGACGTTGAGGAAAACCCA GGTCCTATGAAACCAGTTTACTTG |  |
|  |  | r37: CAAAGAGAAGTTAGTAGCTCCATAGACTGGAGTAA AG |  |
|  | *P2A-nifH* | f38: CTTTACTCCAGTCTATGGAGCTACTAACTTCTCTTTG |  |
|  |  | r38: CTAATTACATGACTCGAGGTCGACTTACTGACCGG AAGCCTCAGC |  |
|  | 10× His | f39: CATCATCACCATCACCATCACCATCACCATTAAGT CGACCTCGAGTCATG |  |
|  |  | r39: ATGGTGATGGTGATGGTGATGGTGATGATGCTGAC CGGAAGCCTCAGC |  |
| *nifU-T2A-nifS-P2A-nifH* | *nifU-T2A* | f36: GTGGATCCCCCGGGCTGCAGGAATTCATGTGGAAC TACTCAGAAAAAG | For construction of plasmid pRS423-USHt |
|  |  | r40: TCTTCGACATCACCACAAGTCAACAAAGAACCTCT ACCTTCGTTCACAGCTGCAGGAGC |  |
|  | *T2A-nifS-P2A* | f41: CTTTGTTGACTTGTGGTGATGTCGAAGAGAACCCTG GTCCAATGAAACCAGTTTACTTG |  |
|  |  | r37: CAAAGAGAAGTTAGTAGCTCCATAGACTGGAGTAA AG |  |
|  | *P2A-nifH* | f38: CTTTACTCCAGTCTATGGAGCTACTAACTTCTCT TTG |  |
|  |  | r38: CTAATTACATGACTCGAGGTCGACTTACTGACCGGA AGCCTCAGC |  |
|  | 10× His | f39: CATCATCACCATCACCATCACCATCACCATTAAGT CGACCTCGAGTCATG |  |
|  |  | r39: ATGGTGATGGTGATGGTGATGGTGATGATGCTGA CCGGAAGCCTCAGC |  |

**Table S4.** Plasmids used in this study

| Plasmids | Relevant characteristics | Reference or source |
| --- | --- | --- |
| pUCE-c1 | A pUC57 derivative carrying *nifB*-P2A-*nifH*-T2A-*nifD*-E2A-*nifK* fusion | GenScript Co. China |
| pUCE-c2 | A pUC57 derivative carrying *nifE*-F2A-*nifN*-T2A-*nifX*-F2A-*hesA*-P2A-*nifV* fusion | GenScript Co. China |
| pRN5101 | A 8.09 kb shuttle vector between *B. subtilis* and *E. coli* with a temperature-sensitive region; Amp^r^, Em^r^ | (Villafane R, 1987) |
| pGBKT7 | A yeast two-hybrid vector for expressing proteins fused to the *GAL4* DNA-binding domain (BD), TRP1, Km^r^ | TaKaRa Co. China |
| pGADT7 | A yeast two-hybrid vector for expressing proteins fused to the *GAL4* activation domain (AD), LEU2, Amp^r^ | TaKaRa Co. China |
| pGBKT7-AD | pGBKT7 derivative, carrying *GAL4* activation domain (AD) , Km^r^ | This study |
| pGBKT7-BH-AD | A pGBKT7 derivative carrying *nifB*-*nifH*-AD fusion | This study |
| PGBKT7-BP2AH-AD | A pGBKT7 derivative carrying *nifB*-P2A-*nifH*-AD fusion | This study |
| PGBKT7-BT2AH-AD | A pGBKT7 derivative carrying *nifB*-T2A-*nifH*-AD fusion | This study |
| PGBKT7-BE2AH-AD | A pGBKT7 derivative carrying *nifB*-E2A-*nifH*-AD fusion | This study |
| PGBKT7-BF2AH-AD | A pGBKT7 derivative carrying *nifB*-F2A-*nifH*-AD fusion | This study |
| pRN5101-proH | A pRN5101 derivative carrying *Pnif-nifH* fusion | This study |
| pRN5101-Ptail | A pRN5101 derivative carrying *Pnif-nifH-*P2Atail fusion | This study |
| pRN5101-Ttail | A pRN5101 derivative carrying *Pnif-nifH-*T2Atail fusion | This study |
| pRN5101-Etail | A pRN5101 derivative carrying *Pnif-nifH-*E2Atail fusion | This study |
| pRN5101-Ftail | A pRN5101 derivative carrying *Pnif-nifH-*F2Atail fusion | This study |
| pRS423 | A yeast expression vector with a 2µ replicon and a *HIS3* selecting marker in yeast. pBR322 replicon; Ampr | (Liu et al., 2019) |
| pRS423-*GAL1p* | A pRS423 derivative carrying inducible promoter *GAL1*, *CYC1* terminator and *HIS3* selecting marker in yeast, pBR322 replicon; Amp^r^ | (Liu et al., 2019) |
| pRS423-BH | A pRS423 derivative carrying *nifB-nifH* fusion fusion | This study |
| pRS423-BP2AH | A pRS423 derivative carrying *nifB*-P2A-*nifH* fusion | This study |
| pRS423-BT2AH | A pRS423 derivative carrying *nifB*-T2A-*nifH* fusion | This study |
| pRS423-BE2AH | A pRS423 derivative carrying *nifB*-E2A-*nifH* fusion | This study |
| pRS423-BF2AH | A pRS423 derivative carrying *nifB*-F2A-*nifH* fusion | This study |
| pRS423-USHp | A pRS423 derivative carrying *nifU*-P2A-*nifS*-P2A-*nifH* fusion | This study |
| pRS423-USHt | A pRS423 derivative carrying *nifU*-T2A-*nifS*-P2A-*nifH* fusion | This study |
| pRS423-c1 | A pRS423 derivative carrying *nifB*-P2A-*nifH*-T2A-*nifD-*E2A*-nifK* fusion | This study |
| pRS424-c2 | A pRS424 derivative carrying *nifE*-F2A-*nifN*-T2A-*nifX*-F2A-*hesA*-P2A-*nifV* fusion | This study |

**References**

Liu, X., Wang, M., Song, Y., Li, Y., Liu, P., Shi, H., Li, Y., Hao, T., Zhang, H., and Jiang, W., et al. (2019). Combined assembly and targeted integration of multigene for nitrogenase biosynthetic pathway in Saccharomyces cerevisiae. *ACS Synth. Biol.* **8**: 1766-1775.

Shao, Z., Zhao, H., and Zhao, H. (2009). DNA assembler, an *in vivo* genetic method for rapid construction of biochemical pathways. *Nucleic. Acids. Res.* **37**: e16.

Villafane R, Bechhofer DH, Narayanan CS, Dubnau D. (1987) Replication control genes of plasmid pE194. *J. Bacteriol.* **169**: 4822-4829.

Wang, L., Zhang, L., Liu, Z., Zhao, D., Liu, X., Zhang, B., Xie, J., Hong, Y., Li, P., and Chen, S., et al. (2013). A minimal nitrogen fixation gene cluster from *Paenibacillus* sp. WLY78 enables expression of active nitrogenase in *Escherichia coli*. *PLOS Genet.* **9**: e1003865.

Wang T, Zhao X, Shi H, Sun L, Li Y, Li Q, Zhang H, Chen S, Li J. (2018) Positive and negative regulation of transferred *nif* genes mediated by indigenous GlnR in Gram-positive *Paenibacillus polymyxa*. *PLoS Genet.* **14**: e1007629.
